# CRISPR–Cas9 Screening Identifies KRAS-Induced COX2 as a Driver of Immunotherapy Resistance in Lung Cancer

**DOI:** 10.1158/0008-5472.CAN-23-2627

**Published:** 2024-04-18

**Authors:** Jesse Boumelha, Andrea de Castro, Nourdine Bah, Hongui Cha, Sophie de Carné Trécesson, Sareena Rana, Mona Tomaschko, Panayiotis Anastasiou, Edurne Mugarza, Christopher Moore, Robert Goldstone, Phil East, Kevin Litchfield, Se-Hoon Lee, Miriam Molina-Arcas, Julian Downward

**Affiliations:** 1 Oncogene Biology Laboratory, Francis Crick Institute, London, United Kingdom.; 2 Bioinformatics and Biostatistics, Francis Crick Institute, London, United Kingdom.; 3 Division of Hematology-Oncology, Department of Medicine, Samsung Medical Center, Sungkyunkwan University School of Medicine, Seoul, Republic of Korea.; 4 Cancer Research UK Lung Cancer Centre of Excellence, University College London Cancer Institute, London, United Kingdom.

## Abstract

Oncogenic KRAS impairs antitumor immune responses. As effective strategies to combine KRAS inhibitors and immunotherapies have so far proven elusive, a better understanding of the mechanisms by which oncogenic KRAS drives immune evasion is needed to identify approaches that could sensitize KRAS-mutant lung cancer to immunotherapy. *In vivo* CRISPR–Cas9 screening in an immunogenic murine lung cancer model identified mechanisms by which oncogenic KRAS promotes immune evasion, most notably via upregulation of immunosuppressive COX2 in cancer cells. Oncogenic KRAS potently induced COX2 in both mouse and human lung cancer, which was suppressed using KRAS inhibitors. COX2 acted via prostaglandin E2 (PGE2) to promote resistance to immune checkpoint blockade (ICB) in lung adenocarcinoma. Targeting COX2/PGE2 remodeled the tumor microenvironment by inducing proinflammatory polarization of myeloid cells and influx of activated cytotoxic CD8^+^ T cells, which increased the efficacy of ICB. Restoration of COX2 expression contributed to tumor relapse after prolonged KRAS inhibition. These results provide the rationale for testing COX2/PGE2 pathway inhibitors in combination with KRAS^G12C^ inhibition or ICB in patients with KRAS-mutant lung cancer.

**Significance:**

COX2 signaling via prostaglandin E2 is a major mediator of immune evasion driven by oncogenic KRAS that promotes immunotherapy and KRAS-targeted therapy resistance, suggesting effective combination treatments for KRAS-mutant lung cancer.

## Graphical Abstract

**Figure:**
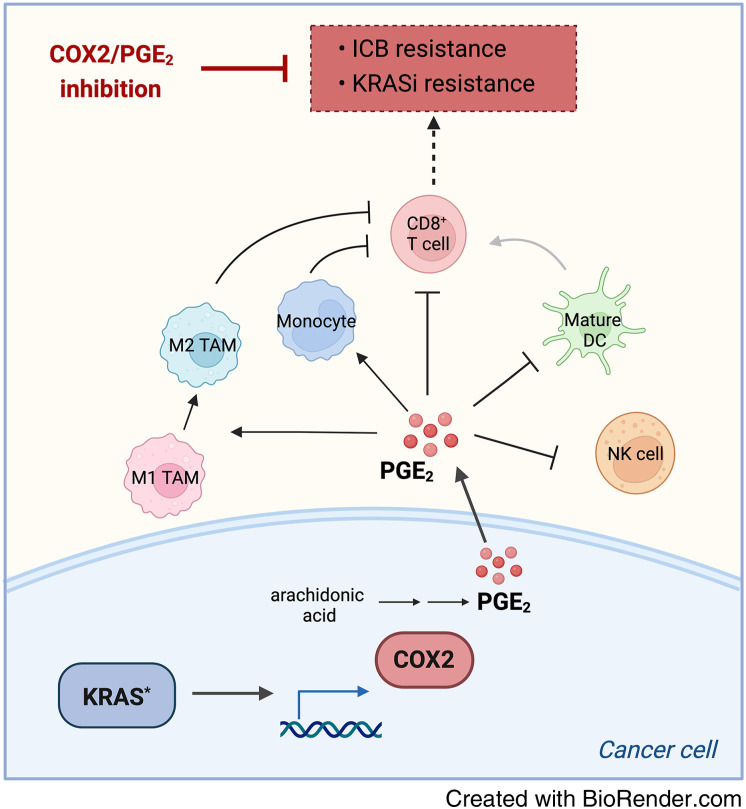

## Introduction

Despite improvements in systemic therapies, lung adenocarcinoma remains the most common cause of cancer-related deaths worldwide (1). Immune checkpoint blockade (ICB), which can reinvigorate antitumor immunity, has shown remarkable clinical success in multiple cancer types (2), including lung adenocarcinoma (3), achieving durable responses in a subset of patients. mAbs targeting the immunosuppressive PDL1/PD1 axis have become the standard of care for patients with lung adenocarcinoma, either as a monotherapy (4) or in combination with chemotherapy (5). However, only a fraction of patients benefit from ICB, highlighting the need for combination strategies that will broaden responses to current immunotherapies. Recent clinical efforts, such as the SKYSCRAPER-01 trial combining PDL1 blockade with an anti-TIGIT Ab, have failed to lead to improved responses in lung cancer, and there is a need to better understand the mechanisms of immune evasion in order to design rational combination strategies that are more likely to provide clinical benefit.

Mutations in the oncogene KRAS drive tumorigenesis in 30% of lung adenocarcinoma cases (6). However, the development of inhibitors that directly target KRAS has been notoriously challenging (7). A major breakthrough was achieved with the recent development of mutant-specific KRAS^G12C^ inhibitors (8), which covalently bind to the novel cysteine residue present in nearly half of all patients with KRAS-mutant lung adenocarcinoma (9). This has led to the approval of Amgen’s clinical compound sotorasib for the treatment of locally advanced or metastatic KRAS^G12C^-mutant lung cancer (10). Although these drugs have mild toxicities and achieve clinical responses in a substantial proportion of patients, as with other targeted therapies, responses are often short-lived as resistance inevitably arises (11).

Accumulating evidence suggests that oncogenic signaling extends beyond the tumor cell compartment and engages with the host stromal and immune compartments. Consequently, oncogenic drivers have been shown to play dominant roles in shaping the tumor immune landscape of different cancers and inhibiting antitumor immune responses (12). Analysis of clinical samples has demonstrated that KRAS mutations are associated with an immunosuppressive tumor microenvironment (TME; ref. 13), and preclinical studies have identified a number of mechanisms by which oncogenic KRAS can drive immune evasion including promoting the expression of numerous immunosuppressive myeloid chemoattractants (14, 15) and immune checkpoint ligands (16). Taken together, these observations provide a rational basis for combining KRAS inhibitors with ICB, and numerous preclinical studies have demonstrated that this combination leads to improved therapeutic responses, at least in immunogenic models (15, 17, 18). However, recent reports of the CodeBreaK 100/101 clinical trial evaluation of sotorasib in combination with anti-PDL1/PD1 Abs have shown serious toxicities (19, 20), casting doubt on the viability of this combination. As an alternative approach, a greater understanding of the mechanisms by which oncogenic KRAS drives immune evasion may identify novel immunotherapy combination strategies that could improve outcomes for patients with KRAS-mutant lung cancer.

Pooled CRISPR screens have been increasingly used to uncover tumor-intrinsic determinants of antitumor immunity, identifying numerous genes that either promote sensitivity or resistance to immune control (21–25). We recently developed a novel immunogenic model of KRAS-mutant lung cancer, allowing for the preclinical study of tumor–immune interactions (17). In this study, we carry out a pooled *in vivo* CRISPR screen in this novel model to interrogate the role of 240 KRAS-regulated genes in controlling antitumor immunity. This identified several genes that increased sensitivity or resistance to antitumor immune responses. Among these, the prostaglandin synthase COX2, responsible for the synthesis of the immunosuppressive molecule prostaglandin E2 (PGE2), was identified as a major driver of immune evasion and resistance to immunotherapy in both mouse and human lung cancer. Targeting of the COX2/PGE2 axis improved the response of KRAS-mutant lung tumors to anti-PD1 therapy by inducing proinflammatory polarization of myeloid cells and enhancing T-cell infiltration and activation. Importantly, oncogenic KRAS signaling was a strong driver of the COX2/PGE2 signaling axis in both mouse and human lung adenocarcinoma, and COX2 inhibition delayed tumor relapse after KRAS^G12C^ inhibition.

## Materials and Methods

### *In vivo* tumor studies

All animal studies were approved by the Ethics Committee of The Francis Crick Institute and conducted according to local guidelines and UK Home Office regulations under the Animals Scientific Procedures Act 1986.

All transplantation animal experiments were carried out using 8- to 12-week-old C57BL/6J mice. For orthotopic experiments, mice were injected with 1.5 × 10^5^ KPAR or KPAR^G12C^ cells intravenously into the tail vein. The mice were euthanized when they displayed signs of ill health or exceeded 15% weight loss. For subcutaneous experiments, 1 × 10^6^ 3LL ΔNRAS cells were injected subcutaneously into the left flank (1:1 mix with Matrigel). Tumors were measured twice weekly using calipers, and the volume was calculated using the formula 0.5 × length × width^2^. The mice were euthanized when the average tumor dimension exceeded 1.5 cm. *Kras*^LSL-G12D/+^;*Trp53*^fl/fl^ mice (KP) were sourced from the Mouse Models of Human Cancer Consortium and maintained on a pure C57BL/6 background. Tumors were induced by intratracheal intubation of 1 × 10^6^ adenoviruses expressing Cre recombinase.

For Ab treatments, 200 μg anti-PD1 (clone RMP1-14, Bio X Cell, AB_2927529) or the respective IgG control (AB_1107769) was administered via i.p. injection twice weekly for a maximum of 3 weeks. For drug treatments, 50 mg/kg MRTX849 (MedChemExpress), 1.3 mg/kg trametinib (LC Laboratories), 16.6 mg/kg linsitinib (Astellas), 1.6 mg/kg everolimus (LC Laboratories), 30 mg/kg celecoxib (LC Laboratories), 100 mg/kg TPST-1495 (kindly provided by Tempest Therapeutics), or their respective vehicles were administered daily or twice daily via oral gavage for the stated duration. MRTX849 was prepared in 10% Captisol diluted in 50 mmol/L citrate buffer (pH 5.0); trametinib, everolimus, and linsitinib in 0.5% methylcellulose/0.2% Tween 80; celecoxib in a mixture of 10% DMSO, 50% polyethylene glycol 400, and 40% water; and TPST-1495 in 0.5% methylcellulose. For orthotopic experiments involving targeted therapies, mice were randomized into groups, and treatments initiated once lung tumors reached an average volume of 1.5 mm^3^, as detected by micro-CT.

For depletion experiments, mice received 200 µg anti-CD8 (2.43, AB_1125541) and/or 200 µg anti-NK1.1 (PK136, AB_1107737) via i.p. injection 1 and 3 days before tumor cell transplantation followed by once weekly for the duration of the experiment. Depletion was confirmed by flow cytometry using anti-CD49b-AF488 (DX5, BioLegend, AB_492879) and anti-Nkp46-BV421 (29A1.4, BioLegend, AB_ 2737837) for NK cells and anti-CD8-PE (53-6.7, BD Biosciences, AB_394571) for CD8^+^ T cells.

### Single-guide RNA library generation

Single-guide RNAs (sgRNA) were designed to target upstream of the first functional domain of each gene. This was identified for each gene using the coding sequence of the principal isoform (APPRIS database) for each transcript and protein domain annotation from the Pfam database. The top five ranked sgRNAs, based on on-target and off-target activity, were chosen. Some genes could only be targeted by four sgRNAs because of their short length. A total of 1,191 sgRNAs, targeting 240 genes, were designed using the GPP sgRNA designer (http://portals.broadinstitute.org/gpp/public/analysis-tools/sgrna-design). Fifty nontargeting sgRNAs were also used from the mouse GeCKO v2 library. Annealed oligonucleotides corresponding to each sgRNA were combined into 10 different pools, each containing 125 sgRNAs and five nontargeting controls, and cloned into a lentiviral vector (pLenti_BSD_sgRNA). Lentiviral libraries were produced using each of the 10 pools and stored at −80°C. The sgRNA sequences used in the library are given in Supplementary Table S1.

### *In vivo* CRISPR screening

A total of 1 × 10^6^ KPAR iCas9 cells were infected with each of the 10 lentivirus pools at a multiplicity of infection (MOI) of 0.3. Twenty-four hours after infection, cells were selected in blasticidin (10 µg/mL, InvivoGen) for 4 days. The selected cells were subsequently expanded *in vitro*, and Cas9 was induced by doxycycline (1 µg/mL, Sigma) for 6 days. Cells were then changed into normal media for 2 days before being orthotopically transplanted into mice. A total of 1.25 × 10^5^ library-transduced cells were injected intravenously into the tail vein of wild-type (WT) C57BL/6J mice and *Rag2*^*−/−*^; *Il2rg*^*−/−*^ mice. In parallel, library-transduced cells were cultured *in vitro* at a library representation of >2000× for the same time period as the *in vivo* experiment. Tumors were harvested 3 weeks after transplantation, and genomic DNA was extracted from tumors using the Gentra Puregene DNA extraction kit (QIAGEN).

Two-step PCR of genomic DNA was performed to amplify the sgRNA sequences and attach sequencing adapters required for Illumina sequencing. sgRNA representation in each sample was measured by sequencing amplicons using an Illumina NextSeq 500. Data analysis was performed by the bioinformatics facility at The Francis Crick Institute. Briefly, reads were initially assigned to each sample using the indexed barcodes and then aligned to one of the possible sgRNAs in the library. Reads were normalized to total read counts per sample using MAGeCK and log_2_-fold change between groups calculated. Guides with less than 15,000 reads at the time of library transduction were removed from the analysis.

### Cell lines

KPAR, KPAR^G12C^, and 3LL ΔNRAS cells were generated as previously described (17, 26)*.* CT26^G12C^ cells were kindly provided by Mirati Therapeutics (27). NCI-H23 (CVCL_1547), NCI-H358 (CVCL_1559), NCI-H1792 (CVCL_1495), NCI-H2030 (CVCL_1517), and A549 (CVCL_0023) cells were obtained from The Francis Crick Institute Cell Services facility. Cell lines were cultured in DMEM or RPMI supplemented with FBS (10%), L-glutamine (2 mmol/L), penicillin (100 units/mL), and streptomycin (100 µg/mL). Clonal cell lines were derived by single-cell dilution into 96-well plates in DMEM-F12 supplemented with GlutaMAX, FBS (10%), hydrocortisone (1 µmol/L), EGF (20 ng/mL), and insulin-like growth factor (50 ng/ml). Cell lines were routinely tested for *Mycoplasma* infection and were authenticated by short tandem repeat DNA profiling by The Francis Crick Institute Cell Services facility. For *in vitro* growth experiments, 2 × 10^4^ cells were plated in 96-well plates, and cell confluency was monitored every 3 hours for 5 days using an Incucyte ZOOM system (Essen BioScience).

### *In vitro* treatments

Drugs or cytokines were added to fresh media 24 hours after seeding cells at stated concentrations, and samples were obtained at indicated time points.

### Flow cytometry

Mouse tumors were cut into small pieces and incubated with collagenase type I (1 mg/mL; Thermo Fisher Scientific) and DNase I (50 U/ml; Life Technologies) in Hank’s Balanced Salt Solution for 45 minutes at 37°C. Cells were filtered through 70-μm strainers (Falcon), and red blood cells were lysed using ACK buffer (Life Technologies). Samples were stained with fixable viability dye eFluor870 (BD Horizon) for 30 minutes and blocked with CD16/32 Ab (BioLegend) for 10 minutes before fluorescently labeled Ab staining of surface markers (see Supplementary Table S2; Supplementary Fig. S5A and S5B). Intracellular staining was then performed after fixation using the fixation/permeabilization kit (eBioscience), according to the manufacturer’s instructions. Samples were resuspended in FACS buffer and analyzed using a BD Symphony flow cytometer. Data were analyzed using FlowJo (Tree Star).

For FACS analysis *in vitro*, cells were trypsinized, washed with FACS buffer, and stained with the following Abs: anti–IFNγR-β-chain-PE (MOB-47, BioLegend, AB_313560), anti–H2Db-PE (KH95, BioLegend, AB_313512), or anti–PDL1-PE-Cy7 (10F.9G2, BioLegend, AB_10643573).

### IHC

Tumor-bearing lungs were fixed in 10% neutral buffer formalin for 24 hours and transferred to 70% ethanol. Fixed lungs were processed into paraffin-embedded blocks. Tissue sections were stained with hematoxylin and eosin, using the automated Tissue-Tek Prisma slide stainer. For IHC staining, the sections were boiled in sodium citrate buffer (pH 6.0) for 15 minutes and incubated with the following Abs for 1 hour: anti-CD8 (4SM15, Thermo Fisher Scientific, AB_2572861), anti-NCR1 (EPR23097-35, Abcam, AB_2904203), and anti-Arg1 (D4E3M, Cell Signaling Technology, AB_2800207). Primary Abs were detected using biotinylated secondary Abs and horseradish peroxidase/3-3'-diaminobenzidine (DAB) detection. Slides were imaged using a Leica ZEISS AxioScan.Z1 slide scanner. Tumor-infiltrating immune cells were quantified using QuPath.

### RT-qPCR

RNA was extracted from cell lines or frozen lung tumors using RNeasy kit (QIAGEN). Single tumor nodules were plucked from tumor-bearing lungs. At least two tumors from at least three mice were included per group in each analysis. Frozen tumor samples were homogenized prior to RNA extraction using QIAshredder columns (QIAGEN). cDNA was generated using the Maxima First Strand cDNA synthesis kit (Thermo Fisher Scientific), and qPCR was performed using Applied Biosystems Fast SYBR Green reagents (Thermo Fisher Scientific). mRNA relative quantity was calculated using the ΔΔCT method and normalized to *Sdha*, *Hsp90*, and *Tbp*. For heatmap visualization, relative mRNA expression for each gene was log-transformed and median-normalized. Hierarchical clustering of gene expression data was carried out with the Euclidian distance metric using Morpheus (Broad Institute).

### ELISA assay

Cells were treated as indicated for 48 hours. Conditioned media were collected, and PGE2 concentration was determined using the PGE2 Parameter Assay Kit (R&D), as per the manufacturer’s instructions.

### Enzyme-linked immunosorbent spot assay

A total of 1×10^5^ CD8^+^ T cells were isolated from spleens of tumor-bearing mice using the EasySep Mouse CD8a Positive Selection Kit (STEMCELL Technologies) and pulsed with 1 µmol/L of the ecotropic murine leukemia retrovirus (eMLV) envelope MHC I–restricted peptide (KSPWFTTL). Cells were stimulated for 24 hours in antimouse IFNγ-coated enzyme-linked immunosorbent spot (ELISPOT) plates (BD Biosciences) and developed, according to the manufacturer’s instructions. Spots were quantified using a CTL S6 machine.

### siRNA experiments

Cells were seeded in a six-well plate and reverse-transfected with 50 nmol/L siGENOME siRNA pools targeting mouse *Myc* (Dharmacon) using the DharmaFECT 4 transfection reagent (Dharmacon), according to the manufacturer’s instructions. Twenty-four hours after transfection, cells were treated for 24 hours with trametinib (10 nmol/L). Control cells were mock-transfected (no siRNA) or transfected with siGENOME RISC-Free control siRNA (Dharmacon).

### Micro-CT imaging

Mice were anesthetized by inhalation of isoflurane and scanned using the Quantum GX2 micro-CT imaging system (PerkinElmer). Serial lung images were reconstructed, and tumor volumes were subsequently analyzed using Analyze (AnalyzeDirect), as previously described (28)

### Western blotting

Cells were lysed using 10× cell lysis buffer (Cell Signaling Technology) supplemented with protease and phosphatase inhibitors (Roche). The protein concentration was determined using a BCA protein assay kit (Pierce), and 15 to 20 μg of protein was separated on a 4% to 12% NuPAGE Bis-Tris gel (Life Technologies) followed by transfer to polyvinylidene difluoride membranes. Proteins were detected by Western blotting using the following primary Abs against Flag (M2, Sigma, AB_262044), ERK1/2 (3A7, Cell Signaling Technology, AB_10695739), pERK1/2 (Thr202/Tyr204; 9101, Cell Signaling Technology, AB_331646), Myc (Y69, Abcam, AB_731658), STAT1 (9172, Cell Signaling Technology, AB_2198300), pSTAT1 (T701; 58D6, Cell Signaling Technology, AB_561284), STAT2 (D9J7L, Cell Signaling Technology, AB_2799824), COX2 (D5H5, Cell Signaling Technology, AB_2571729), vinculin (VIN-11-5, Sigma, AB_2877646), and β-actin (8H10D10, Cell Signaling Technology, AB_2242334). Primary Abs were detected using horseradish peroxidase–conjugated secondary Abs and visualized using a standard film. Alternatively, the membranes were incubated with secondary conjugates compatible with IR detection at 700 and 800 nm and scanned using the Odyssey IR imaging system (Odyssey, LI-COR).

### CRISPR–Cas9 knockout

*Ptgs2* ^*−*/*−*^, *Ifngr2*^*−*/*−*^, and *Etv4*^*−*/*−*^ KPAR cell lines were generated by transient transfection of a Cas9–sgRNA plasmid (pX459, Addgene) generated using standard molecular cloning techniques (see Supplementary Table S3 for sgRNA sequences). A total of 3 × 10^5^ cells were seeded in a six-well plate and transfected 24 hours later with 5 µg pX459 plasmid DNA using Lipofectamine 3000 (Thermo Fisher Scientific), according to the manufacturer’s instructions. After 24 hours, cells were selected in puromycin (5 µg/mL, InvivoGen) for 48 hours. After selection, cells were single-cell–cloned by single-cell dilution into 96-well plates. Knockout clones were identified by Western blotting, ELISA, or flow cytometry. *Edn1*^*−*/*−*^ KPAR cells were generated by infecting KPAR iCas9 cells with a pool of five different lentiviruses each encoding a different sgRNA cloned into a lentiviral vector (pLenti_BSD_sgRNA). Cells were infected at a high MOI to maximize the number of lentivirus particles taken up by cells and increase the efficacy of editing. Twenty-four hours after infection, cells were selected in blasticidin (10 µg/mL, InvivoGen) for 4 days, and Cas9 was induced by doxycycline (1 µg/mL, Sigma) for 6 days. As a negative control, cells were infected with a pool of five different lentiviruses each encoding a different nontargeting sgRNA. sgRNAs were designed using the GPP sgRNA designer (http://portals.broadinstitute.org/gpp/public/analysis-tools/sgrna-design).

### Stable cell lines, plasmids, and lentivirus infection

For the generation of the KPAR iCas9 cell line, KPAR cells were infected with the lentiviral vector pCW-Cas9 (Addgene) at a MOI of 0.3. Twenty-four hours after infection, cells were selected in hygromycin (500 µg/mL, InvivoGen) for 7 days. Antibiotic-selected cells were single-cell–cloned by single-cell dilution in 96-well plates. Clones with minimal expression of Cas9 in normal media and robust induction of Cas9 expression after 24 hours treatment with doxycycline (1 µg/mL, Sigma) were identified by Western blotting.

Lentivirus particles were generated by cotransfection of HEK293T cells (CVCL_0063) with the lentiviral vector and packaging plasmids pCMV-VSV-G and pCMV-dR8.2. Forty-eight hours after transfection, the supernatant was collected, filtered through a 0.45-µm filter, and frozen at −80°C. Cells were infected with lentivirus particles by spinfection. Briefly, 1 × 10^6^ cells were plated in a 12-well plate along with 8 µg/mL polybrene (Millipore) and the specific volume of lentivirus depending on the MOI desired. The cells were centrifuged at 1,000 *g* for 2 hours at 33°C. After the spin, 2 mL of media was added, and 16 hours later, cells were trypsinized and plated into six-well plates. Twenty-four hours after spinfection, the cells were selected with appropriate antibiotic and subsequently expanded. MOI was calculated for each lentivirus batch by infecting target cells with different dilutions of lentivirus, as previously described (29).

### Bioinformatic analysis

The Cancer Cell Line Encyclopedia (CCLE) RNA sequencing (RNA-seq) data were obtained from the CCLE repository hosted at the Broad Institute (https://data.broadinstitute.org/ccle_legacy_data). We used the classification of RAS-low and RAS-high, as previously described (30). All The Cancer Genome Atlas (TCGA) RNA-seq gene-level read counts were downloaded using the TCGAbiolinks (TCGAbiolinks_2.8.4) package from Bioconductor (legacy = TRUE). Samples were classified in RAGs using RAS84, as previously shown (30). Raw counts for the TCGA lung adenocarcinoma, CCLE lung cell lines, and lung adenocarcinoma ICB cohort were VST-normalized using the varianceStabilizingTransformation function within DESeq2 (DESeq2_1.20.0) from Bioconductor.

The COX-IS score was calculated as the mean expression (vst estimate) of the COX-IS cancer-promoting genes (*VEGFA*, *CCL2*, *IL8*, *CXCL1*, *CXCL2*, *CSF3*, *IL6*, *IL1B*, and *IL1A*) divided by the mean expression (vst estimate) of the COX-IS cancer-inhibitory genes (*CCL5*, *CXCL9*, *CXCL10*, *CXCL11*, *IL12A*, *IL12B*, *IFNG*, *CD8A*, *CD8B*, *GZMA*, *GZMB*, *EOMES*, *PRF1*, *STAT1*, and *TBX21*), as previously described (31). COX-IS was used to stratify ICB-treated patients with lung adenocarcinoma into top 25% and bottom 25% quartiles, and univariate survival analysis was carried out. Responders were defined as patients with partial or complete response, and nonresponders were defined as patients with stable or progressive disease.

### Statistical analysis

Statistical significance was assessed in Prism 7 (GraphPad Software) using either an unpaired, two-tailed Student *t* test, log-rank test, one-way ANOVA, or two-way ANOVA, as indicated. *P* ≤ 0.05 was considered statistically significant (^∗^, *P* < 0.05; ^∗∗^, *P* < 0.01; ^∗∗∗^, *P* < 0.001; ^∗∗∗∗^, *P* < 0.0001).

### Data availability

Human expression data from human cancer cell lines and patient samples are publicly available in CCLE and TCGA, respectively. Expression data from patients with lung cancer treated with ICB are available from the authors upon request. All other raw data are available upon request from the corresponding authors.

## Results

### *In vivo* CRISPR screen identifies tumor-intrinsic determinants of antitumor immunity

It is challenging to carry out large-scale genome-wide screens *in vivo* while also maintaining a sufficiently high representation of the pooled library, as there is a limit on the number of cells that can be orthotopically transplanted. Instead, a rationally selected, smaller, customized library was used. First, we generated a library of lentiviral vectors encoding sgRNAs targeting 240 genes that are regulated by KRAS in human lung adenocarcinoma. KRAS-regulated genes were identified by differential gene expression analysis of TCGA lung adenocarcinoma samples and lung adenocarcinoma cell lines from the CCLE, which were stratified as having high or low RAS pathway activity using a novel 84-gene RAS transcriptional metasignature derived from multiple RAS pathway signatures (30). Additional genes were identified using RNA-seq data from KRAS^G12C^-mutant lung adenocarcinoma cell lines (H358 and H23) treated with a KRAS inhibitor and immortalized type II pneumocytes expressing an ER-KRAS^G12V^ fusion protein, which can be readily activated by administration of 4-hydroxytamoxifen (15). To carry out the screen, we utilized the immunogenic KPAR cell line, derived from a genetic KRAS^G12D^ p53^−/−^ (KP) lung cancer mouse model, as it stimulates endogenous antitumor immune responses and is partially responsive to immunotherapy (17). Next, we engineered KPAR cells to express Cas9 under a doxycycline-inducible promoter (Supplementary Fig. S1A). An inducible system was chosen as it allows temporal control of editing and circumvents any compounding consequences of Cas9 immunogenicity *in vivo*. Importantly, Cas9 expression was abrogated *in vitro* 48 hours after the removal of doxycycline (Supplementary Fig. S1B) and was not re-expressed *in vivo* (Supplementary Fig. S1C).

**Figure 1.**
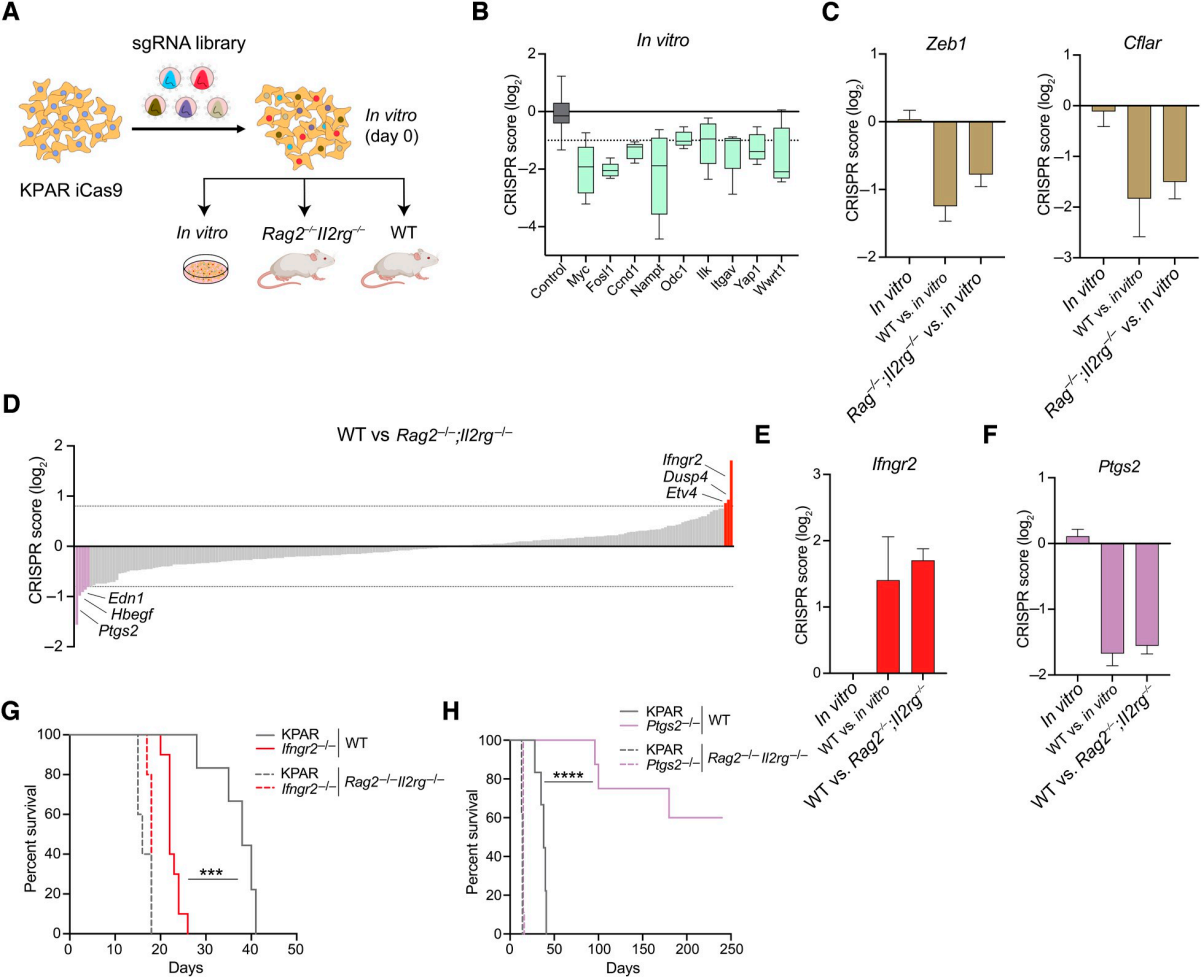
*In vivo* CRISPR–Cas9 screen identifies regulators of antitumor immunity. **A,** Schematic of pooled CRISPR–Cas9 screen. **B,** sgRNAs targeting genes depleted *in vitro* compared with nontarget controls. The CRISPR score is defined as the average log_2_-fold change in abundance of sgRNA reads at day 28 (*in vitro*) vs. day 0 (*in vitro*) for each gene. **C,** sgRNAs targeting *Cflar* and *Zeb1* depleted *in vivo* in immunocompetent and immunodeficient mice. **D,** Average log_2_-fold change in abundance of sgRNA reads for all genes in immunocompetent (WT) vs. *Rag2*^−/−^*;Il2rg*^−/−^ mice. **E** and **F,** Enrichment of sgRNAs targeting *Ifngr2* (**E**) and depletion of sgRNAs targeting *Ptgs2* (**F**) in WT vs. *Rag2*^−/−^*;Il2rg*^−/−^ mice. Data are represented as mean ± SEM for **C**, **E**, and **F**. **G** and **H,** Kaplan–Meier survival of immunocompetent or *Rag2*^−/−^*;Il2rg*^−/−^ mice following orthotopic transplantation with KPAR cells and *Ifngr2*^−/−^ cells (**G**) or *Ptgs2*^−/−^ cells ( *n* = 5–10 per group; **H**). Analysis of survival curves was carried out using the log-rank (Mantel–Cox) test. ***, *P* < 0.001; ****, *P* < 0.0001.

Library-transduced KPAR iCas9 cells were treated with doxycycline for 4 days to allow gene-editing to occur, followed by a 2-day washout period to abrogate the expression of Cas9 before orthotopic transplantation into C57BL/6 (WT) mice or *Rag2*^−*/*−^;*Il2rg*^−*/*−^ mice, which lack T cells, B cells, and NK cells and therefore do not exert antitumor immune responses (Fig. 1A). After 3 weeks, genomic DNA was isolated from tumor-bearing lungs and subjected to deep next-generation sequencing to compare library representation in tumors growing in immunocompetent and immunodeficient mice (Supplementary Table S1). In parallel, next-generation sequencing of genomic DNA from cells passaged *in vitro* was carried out to identify genes that affect cell viability. The analysis of genes targeted by sgRNAs that were depleted *in vitro* identified a number of genes known to affect cell viability in KRAS-mutant tumor cells, including *Myc* (32) and *Fosl1* (33), thereby validating the functionality of the sgRNA library (Fig. 1B). Furthermore, a number of sgRNAs were equally depleted in immunocompetent and immunodeficient mice compared with *in vitro* passaged cells, including those targeting the epithelial–mesenchymal transition regulator *Zeb1* and the antiapoptotic caspase inhibitor c-FLIP (encoded by *Cflar*; Fig. 1C). These genes therefore supported tumor growth *in vivo* by mechanisms independent of antitumor immunity.

By comparing sgRNAs that were depleted or enriched in immunocompetent versus immunodeficient mice, we identified genes that modulate antitumor immune responses (Fig. 1D). The most enriched sgRNAs in immunocompetent mice were targeting a subunit of the IFNγ receptor (*Ifngr2*; Fig. 1E). Furthermore, we also observed enrichment of sgRNAs targeting the Ets transcription factor ETV4 (Supplementary Fig. S1D). Importantly, *Etv4*^−*/*−^ KPAR tumors grew faster than parental tumors in WT mice but not in *Rag2*^−*/*−^;*Il2rg*^−*/*−^ mice (Supplementary Fig. S1E), confirming a role for ETV4 in sensitizing tumors to antitumor immune responses. Indeed, the gene expression analysis of *Etv4*^−*/*−^ KPAR tumors demonstrated that the loss of ETV4 resulted in a drastic downregulation of multiple antitumor immunity genes (Supplementary Fig. S1F). Conversely, the most depleted sgRNAs in immunocompetent mice targeted the prostaglandin synthase COX2, encoded by *Ptgs2* (Fig. 1F). In addition, sgRNAs targeting the secreted protein endothelin 1, encoded by *Edn1*, were also depleted in immunocompetent mice (Supplementary Fig. S1G). Confirming the role of tumor-derived EDN1 in facilitating immune evasion, *Edn1*^−*/*−^ KPAR tumors grew slower than parental tumors specifically in immunocompetent mice (Supplementary Fig. S1H).

### KRAS suppresses antitumor immunity by inhibition of tumor-intrinsic IFNγ signaling

Tumor-intrinsic IFNγ signaling has previously been shown to be required for antitumor immune responses in carcinogen-induced mouse models of cancer (34) and responses to immunotherapy in melanoma (35). To validate the role of tumor-intrinsic IFNγ signaling in antitumor immune responses uncovered in the screen, we generated *Ifngr2*^−/−^ KPAR cell lines using CRISPR–Cas9 (Supplementary Fig. S2A). As expected, *Ifngr2*^−/−^ KPAR cells were insensitive to IFNγ *in vitro* (Supplementary Fig. S2B). Although *Ifngr2*^−/−^ cells grew similarly to parental cells *in vitro* (Supplementary Fig. S2C), they grew faster than parental cells when transplanted into immunocompetent mice (Fig. 1G). In contrast, *Ifngr2*^−/−^ and parental tumors grew at similar rates in immune-deficient *Rag2*^−/−^;*Il2rg*^−/−^ mice. These data were validated using a second clone (Supplementary Fig. S2D), indicating that intact tumor-intrinsic IFNγ signaling is required for effective antitumor immunity.

IFNγ has been shown to have direct antiproliferative effects in human cancer cell lines (35). However, the growth of KPAR cells *in vitro* was unaffected by treatment with IFNα, IFNβ, or IFNγ (Supplementary Fig. S2E). Tumor-intrinsic IFNγ signaling also regulates the expression of antigen presentation machinery and T-cell chemoattractants. *Ifngr2*^−/−^ KPAR cells failed to upregulate IFN signaling molecules, IFN response genes, and antigen presentation machinery genes upon treatment with IFNγ (Supplementary Fig. S2F). Furthermore, *Ifngr2*^−/−^ KPAR cells were unable to upregulate PDL1 or MHC I surface expression in response to IFNγ but remained sensitive to type I (IFNα and IFNβ) interferons (Supplementary Fig. S2G). Consistent with this, CD45^−^ cells, which include tumor and stromal cells, had reduced surface expression of MHC I and PDL1 in *Ifngr2*^−/−^ KPAR tumors (Supplementary Fig. S2H). In agreement with the hypothesis that tumor-intrinsic IFNγ signaling is required for antitumor immunity, *Ifngr2*^−/−^ KPAR tumors had less central memory (CD62L^+^CD44^+^) CD8^+^ T cells, which are important for durable antitumor immune responses (Supplementary Fig. S2I). Furthermore, several genes involved in cytotoxic antitumor immune responses were downregulated in *Ifngr2*^−/−^ tumors (Supplementary Fig. S2J).

Previous studies have demonstrated that oncogenic KRAS regulates tumor-intrinsic IFN pathway gene expression (15). Indeed, treatment of KPAR cells with the MEK inhibitor (MEKi) trametinib or a previously published three-drug KRAS pathway inhibitor combination (26) of trametinib, everolimus, and linsitinib resulted in upregulation of the IFNγ receptor β subunit (Supplementary Fig. S3A). Moreover, the transcriptional upregulation of IFN response genes in KPAR cells treated *in vitro* with IFNγ or IFNα was greatly potentiated by MEKi treatment, validating previous results in other lung cancer models (Supplementary Fig. S3B and S3C). The oncogene Myc acts as a transcriptional suppressor of type I IFN response genes in pancreatic cancer (36), and we have recently shown that Myc mediates KRAS-driven inhibition of IFN response genes in lung cancer (15). Myc expression is often driven by KRAS, and treatment of KPAR cells with MEKi led to rapid downregulation of Myc (Supplementary Fig. S3D). Consistent with its role in suppressing IFN responses, we observed increased expression of IFN genes in Myc-depleted cells treated with IFNγ (Supplementary Fig. S3E). Furthermore, Myc depletion led to increased expression of IFN signaling molecules, which were not further upregulated upon treatment with MEKi (Supplementary Fig. S3F), suggesting that oncogenic KRAS suppresses tumor-intrinsic IFN responses by driving expression of Myc. In summary, these data suggest that KRAS-mediated inhibition of tumor-intrinsic IFNγ responses, which is required for effective antitumor immunity, may contribute to immune evasion in KRAS-mutant lung cancer.

### Tumor-intrinsic COX2 suppresses innate and adaptive antitumor immunity

*Ptgs2* loss was the strongest sensitizer to antitumor immunity in the screen and encodes the enzyme COX2, which is overexpressed in many cancer types. COX2 is responsible for the synthesis of the prostanoid PGE2, which has been shown to suppress antitumor immunity in preclinical models of colorectal cancer and melanoma (37). To validate the results obtained in the screen, we began by generating *Ptgs2*^−/−^ KPAR cell lines using CRISPR–Cas9. Western blotting confirmed the loss of COX2 expression and PGE2 production in *Ptgs2*^−/−^ cells (Supplementary Fig. S4A). We did not observe any difference in the growth of *Ptgs2*^−/−^ cells and parental cells *in vitro* (Supplementary Fig. S4B). However, they grew considerably slower when orthotopically transplanted into immunocompetent mice, which as a result had significantly increased survival, with 60% of mice experiencing complete tumor rejection (Fig. 1H). Importantly, COX2-deficient tumors grew similarly to parental tumors when transplanted into immune-deficient *Rag2*^−*/*−^;*Il2rg*^−*/*−^ mice. This was further validated using a second clone (Supplementary Fig. S4C). Therefore, *Ptgs2*^−*/*−^ cell lines showed no cell-autonomous defects in tumor progression but were instead sensitized to antitumor immune responses, with immunologic rejection occurring in a substantial proportion of mice.

**Figure 2.**
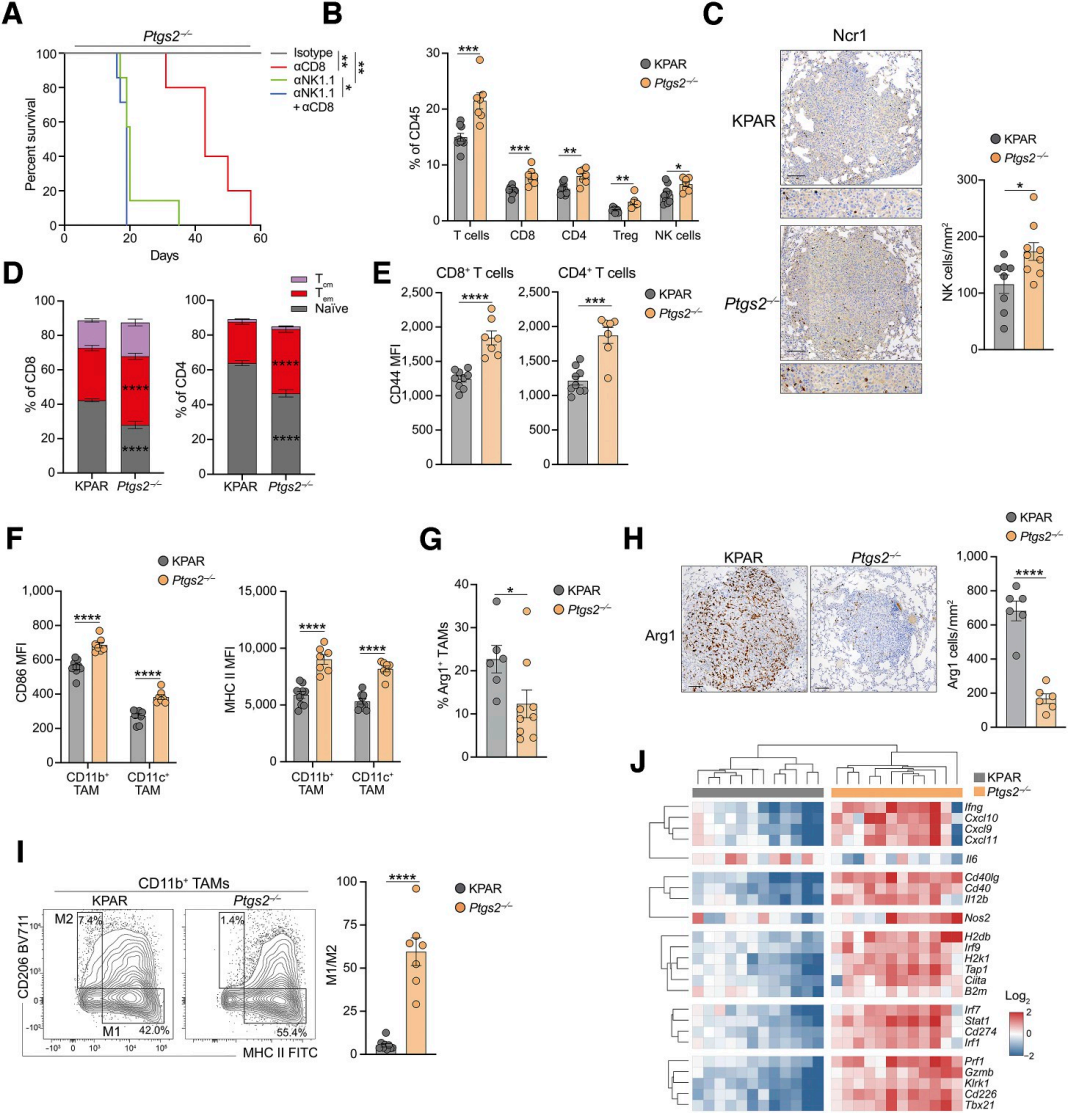
Tumor-intrinsic COX2 suppresses antitumor immunity. **A,** Kaplan–Meier survival of mice treated with 200 μg anti-NK1.1 and/or 200 μg anti-CD8 or corresponding isotype control (*n* = 5–7 per group) after orthotopic transplantation of *Ptgs2*^−*/*−^ cells. Treatment was initiated 3 days before transplantation and was administered once weekly until endpoint. Analysis of survival curves was carried out using the log-rank (Mantel–Cox) test. **B,** Frequency of tumor-infiltrating T-cell populations and NK cells in KPAR and *Ptgs2*^−*/*−^ orthotopic tumors. **C,** Quantification and representative IHC staining for NKp46^+^ NK cells. Scale bar, 100 μm. **D,** Stacked bar plots showing frequency of central memory (T_cm_—CD44^+^CD62L^+^), effector memory (T_em_—CD44^+^CD62L^−^), and naïve (CD44^−^CD62L^+^) CD8^+^ (left) and CD4^+^ (right) T cells. **E,** Surface expression (mean fluorescence intensity, MFI) of CD44 on CD8^+^ (left) and CD4^+^ (right) T cells. **F,** Surface expression (mean fluorescence intensity) of CD86 (left) and MHC II (right) on CD11b^+^ macrophages and CD11c^+^ macrophages. **G,** Percentage of Arg1^+^ CD11b^+^ macrophages. **H,** Quantification and representative IHC staining for the immunosuppressive macrophage marker Arg1. Scale bar, 100 μm. **I,** Representative flow cytometry plots of CD206 and MHC II surface expression on CD11b^+^ macrophages (left) and quantification of the M1/M2 ratio based on the gated populations (right). **J,** Heatmap showing hierarchical clustering of KPAR and Ptgs2^−/−^ tumors based on mRNA expression of antitumor immunity genes assessed by qPCR. Data are represented as mean ± SEM for **B–I**, *n* = 6–9 per group. Symbols represent pooled tumors from individual mice. Statistics were calculated by paired, two-tailed Student *t* test (**B**, **C**, and **E–I**) or two-way ANOVA, FDR 0.05 (**D**). *, *P* < 0.05; **, *P* < 0.01; ***, *P* < 0.001; ****, *P* < 0.0001.

As *Rag2*^−*/*−^;*Il2rg*^−*/*−^ mice lack NK cells, T cells, and B cells, we wanted to decipher the contribution of innate and adaptive immunity to the reduced growth of COX2-deficient tumors in immunocompetent mice. COX2-deficient tumors grew faster in mice treated with Abs depleting either NK cells or CD8^+^ T cells and grew fastest in mice lacking both subsets (Fig. 2A; Supplementary Fig. S4D). Interestingly, tumors grew faster in mice lacking NK cells than in mice lacking CD8^+^ T cells, suggesting that the innate immune response was critical for the impaired growth of COX2-deficient tumors, as previously reported (31). However, no mice survived long term in the absence of CD8^+^ T cells, demonstrating that the combined action of innate and adaptive immunity was required for tumor rejection. NK cells play a critical role in the control of orthotopic lung tumors during tumor cell seeding in the lung. To ensure that the control of COX2-deficient tumors was not exacerbated by the route of injection, we also compared the growth of subcutaneous parental and *Ptgs2*^−*/*−^ tumors. Similar to the orthotopic setting, COX2-deficient subcutaneous tumors grew significantly slower in immunocompetent mice (Supplementary Fig. S4E). Consistent with the role of both innate and adaptive immunity in the rejection of COX2-deficient tumors, flow cytometry showed increased frequencies of CD8^+^ T cells and NK cells as well as CD4^+^ T cells and regulatory T cells (Treg) in orthotopic *Ptgs2*^−*/*−^ lung tumors (Fig. 2B; Supplementary Fig. S5A and S5B). Increased infiltration of COX2-deficient tumors by NK cells was confirmed by IHC (Fig. 2C). We have previously shown that reactivation of the endogenous eMLV in KPAR tumors drives antitumor T-cell responses (17). Indeed, IFNγ ELISPOT showed that *Ptgs2*^−*/*−^ tumors induced an expansion of eMLV-specific CD8^+^ T cells, validating the contribution of T cells to the rejection of *Ptgs2*^−*/*−^ tumors (Supplementary Fig. S6A).

Further flow cytometry analysis revealed drastic changes in the phenotype of both myeloid and adaptive immune subtypes within COX2-deficient tumors. T cells infiltrating *Ptgs2*^−*/*−^ tumors were more activated, with higher frequencies of effector memory (CD44^+^CD62L^−^) CD8^+^ and CD4^+^ T cells (Fig. 2D), mirrored by fewer naïve (CD44^−^CD62L^+^) T cells, an increased frequency of T cells expressing checkpoint receptors (Supplementary Fig. S6B), and upregulation of the activation markers CD44 and CD69 (Fig. 2E; Supplementary Fig. S6C). T cell–mediated immune responses require priming by CD103^+^ dendritic cells (DC) both in lymph nodes and within tumors. Tumor-derived prostaglandins have previously been reported to prevent the accumulation of CD103^+^ DCs into the TME (37); however, we did not observe increased CD103^+^ DCs in *Ptgs2*^−*/*−^ tumors (Supplementary Fig. S6D). Despite this, we observed increased expression of the costimulatory molecule CD86 and MHC II on CD103^+^ DCs (Supplementary Fig. S6E). Similarly, the loss of COX2 did not alter the frequency of tumor-associated macrophages (TAM; Supplementary Fig. S6F) but induced proinflammatory polarization of both CD11b^+^ and CD11c^+^ TAMs with increased expression of both CD86 and MHC II (Fig. 2F; Supplementary Fig. S6G) as well as decreased expression of the M2 markers arginase and CD206 (Fig. 2G and H; Supplementary Fig. S6H). Indeed, when assessing the frequency of M1 (MHC II^+^CD206^−^) and M2 (MHC II^−^CD206^+^) CD11b^+^ TAMs, we observed a dramatic increase in the M1/M2 ratio in COX2-deficient tumors (Fig. 2I). *Ptgs2*^−/−^ tumors also had fewer immunosuppressive Ly6C^+^ monocytes (Supplementary Fig. S6I). In addition, several myeloid subtypes, including CD11b^+^ TAMs, CD103^+^ DCs, CD11b^+^ DCs, and neutrophils, exhibited increased PDL1 expression (Supplementary Fig. S6J), characteristic of a T cell–inflamed TME. Gene expression analysis resulted in distinct clustering of parental KPAR and *Ptgs2*^−/−^ tumors with COX2 loss, leading to increased expression of genes encoding Th1 cytokines (*Ifng*, *Tnfa*, and *Cxcl9*), cytotoxicity genes (*Gzmb* and *Prf1*), IFN response genes (*Irf9*, *B2m*, and *Stat1*), and decreased expression of the immunosuppressive cytokine IL6 (Fig. 2J). Taken together, these data suggest that loss of tumor-intrinsic expression of COX2 results in a remodeling of the TME with increased recruitment of effector cells and polarization of both innate and adaptive immune subsets toward a proinflammatory phenotype, resulting in enhanced antitumor immunity and greater tumor control.

### COX2/PGE2 signaling drives resistance to ICB in mouse and human lung adenocarcinoma

**Figure 3.**
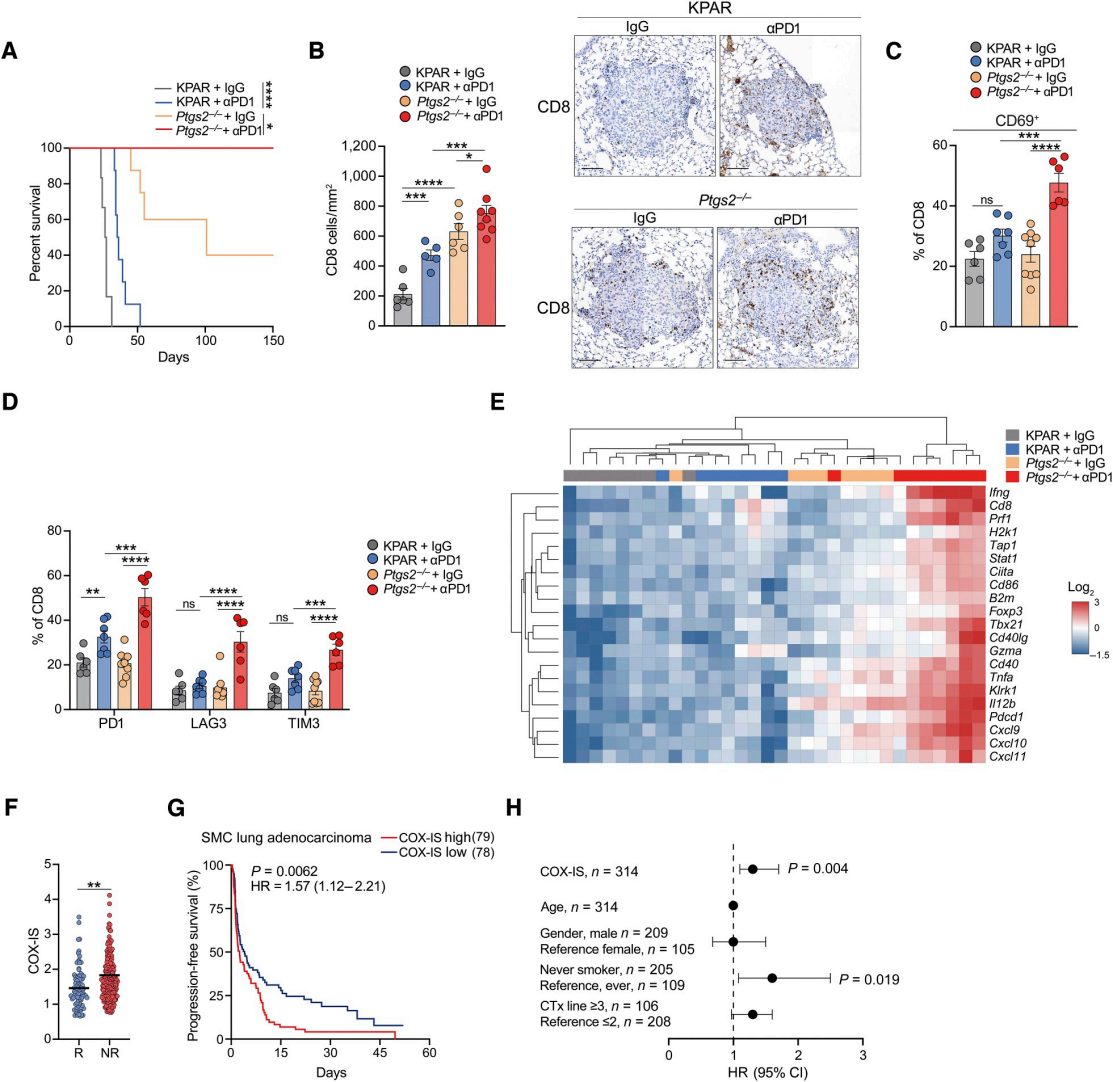
COX2/PGE2 signaling hinders response to ICB in mouse and human lung adenocarcinoma. **A,** Kaplan–Meier survival of mice treated intraperitoneally with 200 μg anti-PD1 after orthotopic transplantation of KPAR or *Ptgs2*^−*/*−^ cells, *n* = 6–8 per group. Analysis of survival curves was carried out using the log-rank (Mantel–Cox) test. **B,** Quantification and representative IHC staining of CD8^+^ T cells in KPAR or *Ptgs2*^−*/*−^ orthotopic tumors on day 7 after treatment with anti-PD1 or corresponding isotype control (IgG). Scale bar, 100 μm. **C,** Percentage of CD69^+^ CD8^+^ T cells in KPAR or *Ptgs2*^−*/*−^ tumors treated as shown in **B**. **D,** Frequency of PD1^+^, LAG3^+^, and TIM3^+^ CD8^+^ T cells in KPAR or *Ptgs2*^−*/*−^ tumors treated as shown in **B**. **E,** Heatmap showing hierarchical clustering of KPAR or *Ptgs2*^−*/*−^ tumors treated as shown in **B** based on mRNA expression of antitumor immunity genes assessed by qPCR. **F,** Baseline COX-IS levels in responder (R) and nonresponder (NR) ICB-treated patients with lung adenocarcinoma. **G,** Progression-free survival of patients with lung adenocarcinoma treated with ICB, stratified into highest and lowest quartile based on COX-IS expression. **H,** Multivariate Cox regression analysis for the indicated variables in patients with lung adenocarcinoma following ICB treatment. CTx, chemotherapy. Error bars represent 95% confidence interval (CI) boundaries. Data are represented as mean ± SEM for **B–D**, *n* = 5–9 per group. Statistics were calculated using two-tailed Student *t* test (**F**) or one-way ANOVA, FDR 0.05 (**B–D**). ns, not significant; *, *P* < 0.05; **, *P* < 0.01; ***, *P* < 0.001; ****, *P* < 0.0001.

Given that tumor-intrinsic COX2 acts as a major driver of immune evasion in KPAR tumors, we wanted to assess whether it also contributed to resistance to ICB. Mice were orthotopically transplanted with parental KPAR or *Ptgs2*^−/−^ cells and treated with anti-PD1. Although parental KPAR tumors were partially responsive to anti-PD1, COX2-deficient tumors were significantly more sensitive to PD1 blockade with all ICB-treated mice bearing *Ptgs2*^−/−^ lung tumors surviving long term (Fig. 3A). IHC revealed that similar to ICB-treated KPAR tumors, *Ptgs2*^−/−^ lung tumors were more infiltrated by CD8^+^ T cells (Fig. 3B), confirming our observation via flow cytometry (Fig. 2B); however, the greatest increase was seen in ICB-treated *Ptgs2*^−/−^ tumors. Furthermore, flow cytometry analysis demonstrated that PD1 blockade only led to increased activation of CD8^+^ T cells in *Ptgs2*^−/−^ lung tumors (Fig. 3C). In addition, ICB-treated *Ptgs2*^−/−^ lung tumors showed the greatest expansion of effector memory CD8^+^ T cells (Supplementary Fig. S7A) and upregulation of checkpoint molecules (Fig. 3D). Interestingly, anti-PD1 treatment also led to increased NK cell infiltration in *Ptgs2*^−/−^ lung tumors, which did not occur in KPAR tumors (Supplementary Fig. S7B). Furthermore, gene expression analysis revealed that anti-PD1 induced robust expression of antitumor immunity genes only in COX2–deficient lung tumors (Fig. 3E; Supplementary Fig. S7C). Together these data suggest that tumor-intrinsic COX2 promotes resistance to ICB by preventing the stimulation of antitumor immunity in response to PD1 blockade.

To determine whether COX2/PGE2 signaling also affected the clinical response of patients with lung cancer to immunotherapy, we examined the expression of a previously published COX2-associated inflammatory gene expression signature (COX-IS; ref. 31) in a cohort of patients with lung adenocarcinoma treated with anti-PDL1/PD1 for which baseline expression data were available (38). Importantly, expression of the COX-IS was significantly higher in patients with lung adenocarcinoma who did not respond to ICB (Fig. 3F). Furthermore, higher COX-IS expression was associated with significantly worse progression-free survival following ICB (Fig. 3G) and was also predictive of outcome independent of age, gender, smoking status, and previous lines of therapy (Fig. 3H). These results support the notion, as suggested by the mouse model, that the COX2/PGE2 axis drives immunosuppression and hinders response to ICB in human lung adenocarcinoma.

### Inhibition of the COX2/PGE2 axis delays tumor growth and synergizes with ICB

**Figure 4.**
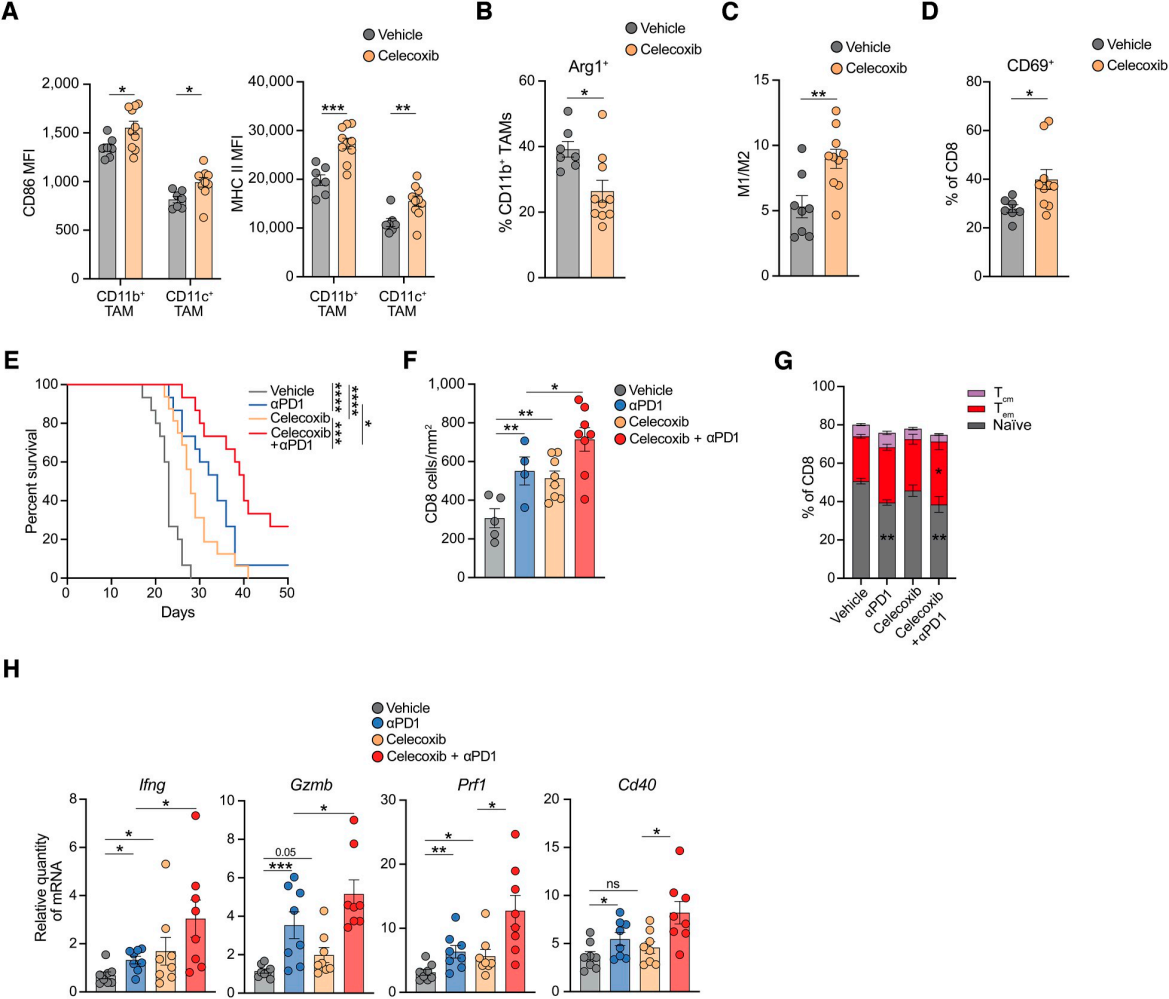
COX2 inhibition enhances the efficacy of immunotherapy. **A,** Surface expression [mean fluorescence intensity (MFI)] of CD86 (left) and MHC II (right) on CD11b^+^ macrophages and CD11c^+^ macrophages in KPAR tumors treated for 7 days with 30 mg/kg celecoxib. **B–D,** Percentage of Arg1^+^ CD11b^+^ macrophages (**B**), quantification of M1/M2 macrophages (**C**), and frequency of CD69^+^ CD8^+^ T cells (**D**) in KPAR tumors treated as shown in **A**. **E,** Kaplan–Meier survival of mice treated intraperitoneally with 200 μg anti-PD1 and/or daily oral gavage of 30 mg/kg celecoxib after orthotopic transplantation of KPAR cells. Daily celecoxib treatment was initiated on day 7 and anti-PD1 began on day 10 and was administered twice weekly for a maximum of 3 weeks. Data from two independent experiments, *n* = 15–16 per group. Analysis of survival curves was carried out using the log-rank (Mantel–Cox) test. **F,** Quantification of CD8^+^ T cells by IHC in KPAR tumors treated for 7 days with celecoxib and/or anti-PD1. **G,** CD8^+^ T-cell phenotypes in KPAR tumors treated as shown in **F**. T_cm_, central memory CD8^+^ T cells (CD44^+^CD62L^+^); T_em_, effector memory CD8^+^ T cells (CD44^+^CD62L^−^). **H,** mRNA expression by qPCR of antitumor immunity genes in KPAR tumors treated as shown in **F**. Data are represented as mean ± SEM (**A–D** and **F–H**), *n* = 5–10 per group. Samples were analyzed using unpaired, two-tailed Student *t* test (**A–D**), one-way ANOVA, FDR 0.05 (**F** and **H**), or two-way ANOVA, FDR 0.05 (**G**). ns, not significant; *, *P* < 0.05; **, *P* < 0.01; ***, *P* < 0.001; ****, *P* < 0.0001.

Although genetic deletion of tumor-intrinsic COX2 resulted in a drastic repolarization of the TME, increased tumor control, and sensitization to ICB, we next sought to assess whether pharmacologic blockade of COX2 could have similar effects. We treated KPAR lung tumor–bearing mice with the COX2-specific inhibitor celecoxib, which was administered by daily oral gavage. As seen in *Ptgs2*^−/−^ lung tumors, treatment of KPAR tumors with celecoxib resulted in polarization of TAMs with upregulation of CD86 and MHC II (Fig. 4A; Supplementary Fig. S8A), decreased expression of arginase (Fig. 4B; Supplementary Fig. S8B), and an increase in the M1/M2 ratio (Fig. 4C), as well as reduced infiltration of Ly6C^+^ monocytes (Supplementary Fig. S8C). Changes in the myeloid compartment were accompanied by the increased activation of CD8^+^ T cells (Fig. 4D).

Importantly, celecoxib significantly extended the survival of KPAR tumor–bearing mice to a similar extent seen with PD1 blockade (Fig. 4E). However, the combination of both celecoxib and anti-PD1 showed superior efficacy compared with either single agent alone. Indeed, both celecoxib and anti-PD1 increased infiltration of tumors with CD8^+^ T cells, which was further increased in the combination treatment arm (Fig. 4F). Furthermore, only the combination treatment led to a significant expansion of effector memory CD8^+^ T cells (Fig. 4G) and upregulation of checkpoint molecules on both CD8^+^ and CD4^+^ T cells (Supplementary Fig. S8D). Combination treatment also induced the highest levels of PDL1 on several myeloid cell types (Supplementary Fig. S8E). This could be due to elevated levels of IFNγ (Fig. 4H), which was significantly upregulated in the combination treatment arm along with other antitumor immunity genes.

**Figure 5.**
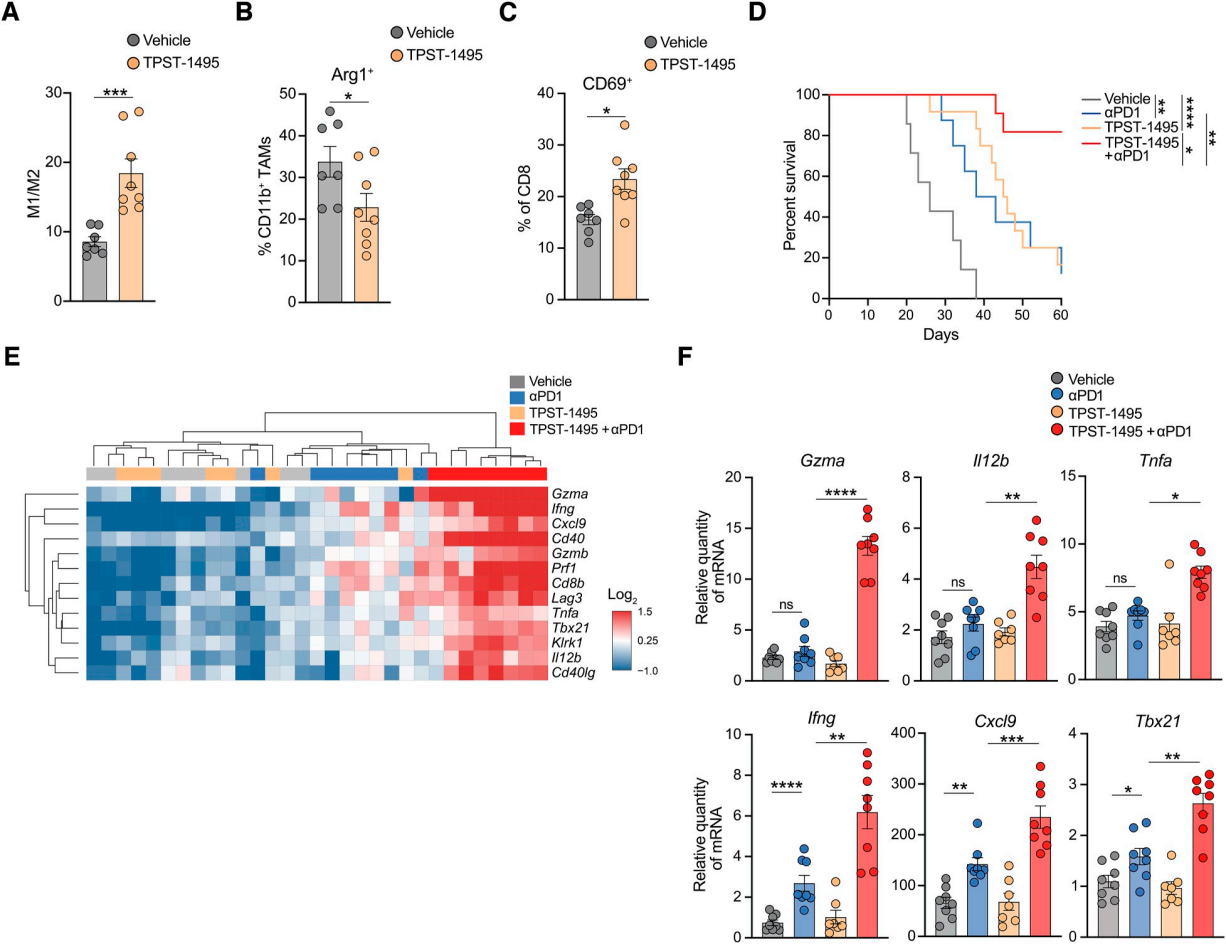
Dual inhibition of EP2 and EP4 synergizes with ICB. **A–C,** M1/M2 macrophage ratio (**A**), percentage of Arg1^+^ CD11b^+^ TAMs (**B**), and percentage of CD69^+^ CD8^+^ T cells (**C**) in KPAR tumors treated twice daily for 7 days with 100 mg/kg TPST-1495. **D,** Kaplan–Meier survival of mice treated intraperitoneally with 200 μg anti-PD1 and/or twice daily oral gavage of 100 mg/kg TPST-1495 after orthotopic transplantation of KPAR cells, *n* = 8–12 mice per group. TPST-1495 treatment was initiated on day 7 and anti-PD1 began on day 10 and was administered twice weekly for a maximum of 3 weeks. Analysis of survival curves was carried out using the log-rank (Mantel–Cox) test. **E,** Heatmap showing hierarchical clustering of KPAR tumors treated for 7 days with TPST-1495 and/or anti-PD1 based on mRNA expression of antitumor immunity genes assessed by qPCR. **F,** mRNA expression by qPCR of immune-related genes in KPAR tumors treated as shown in **E**. Data are represented as mean ± SEM for **A–C** and **F**, *n* = 7–8 mice per group. Statistics were calculated using unpaired, two-tailed Student *t* test (**A–C**) or one-way ANOVA, FDR 0.05 (**F**). ns, not significant; *, *P* < 0.05; **, *P* < 0.01; ***, *P* < 0.001; ****, *P* < 0.0001.

In the clinic, celecoxib has been associated with increased cardiovascular risk (39), which prompted us to explore other therapeutic options to target this immunosuppressive axis. Immune cells express four receptors for PGE2, EP1 to EP4. However, numerous studies have demonstrated that EP2 and EP4 receptors are the primary mediators of the COX2/PGE2 immunosuppressive axis (40, 41). We therefore treated tumor-bearing mice with a novel dual EP2–EP4 antagonist, TPST-1495 (42). As seen with celecoxib, dual EP2–EP4 inhibition led to a significant increase in the M1/M2 macrophage ratio (Fig. 5A) with reduced expression of arginase (Fig. 5B), as well as increased activation of CD8^+^ T cells (Fig. 5C). Indeed, TPST-1495 increased the survival of tumor-bearing mice similarly to PD1 blockade (Fig. 5D). The combination of TPST-1495 and anti-PD1 also significantly extended the survival of mice compared with either monotherapy, with greater synergy compared with the combination of celecoxib and anti-PD1. Furthermore, gene expression analysis revealed distinct clustering of tumors treated with the combination with potent induction of a proinflammatory transcriptional program (Fig. 5E and F). Consistent with this, the combination treatment led to the greatest increase in PDL1 expression on tumor-infiltrating myeloid cells (Supplementary Fig. S8F). In conclusion, these results suggest that pharmacologic inhibition of the COX2/PGE2 axis reverses immunosuppression in the TME, promoting adaptive immunity, which enhances the therapeutic efficacy of ICB.

### COX2 expression is driven by oncogenic KRAS and contributes to tumor relapse after KRAS^G12C^ inhibition

**Figure 6.**
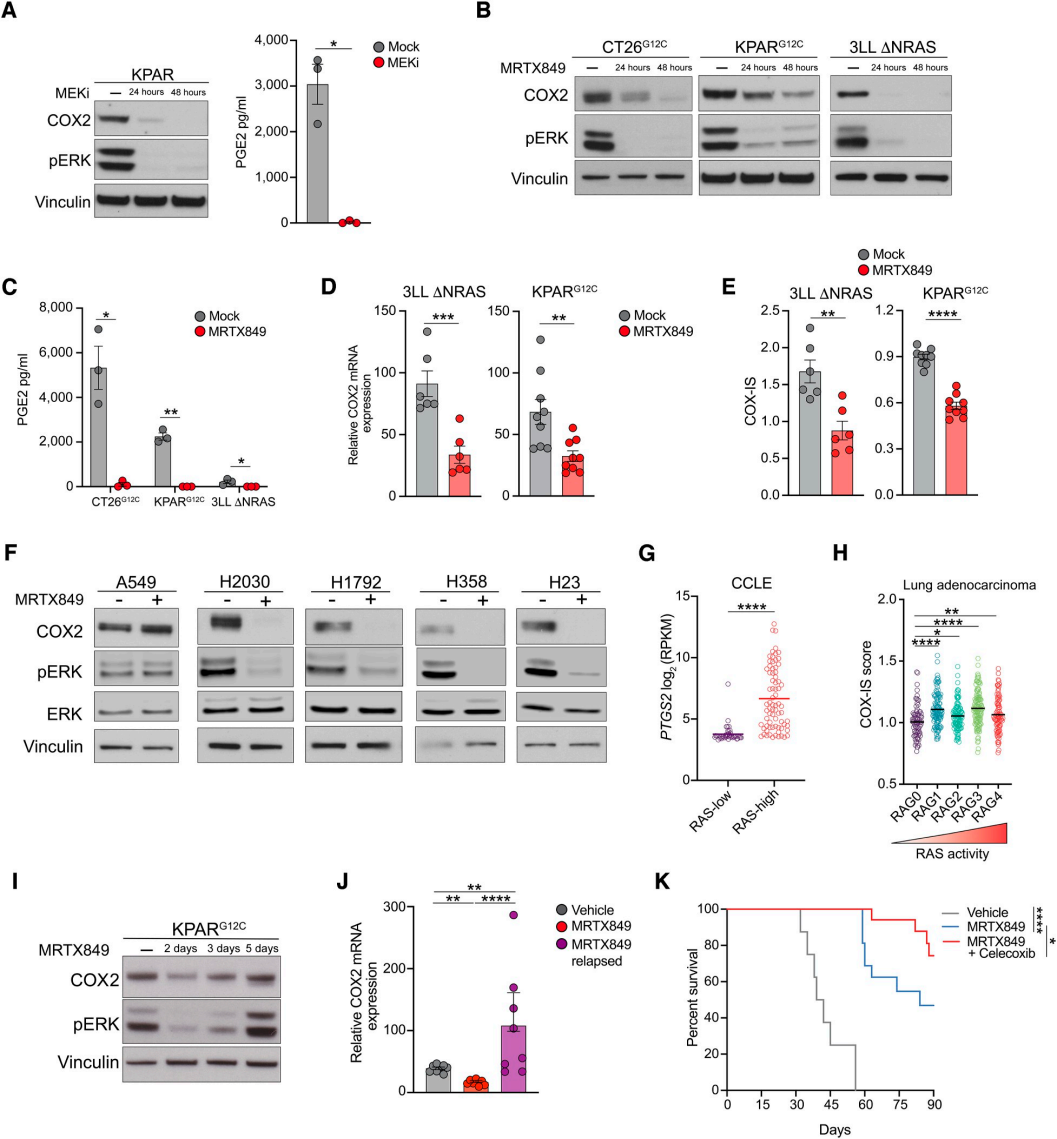
Oncogenic KRAS drives immunosuppressive COX2 expression in lung adenocarcinoma. **A,** Immunoblot for COX2 (left) and ELISA analysis for PGE2 concentration (right) in KPAR cells treated with 10 nmol/L trametinib (MEKi) for 24 hours or 48 hours. **B** and **C,** Immunoblot for COX2 (**B**) and ELISA analysis for PGE2 concentration (**C**) in KRAS^G12C^ mouse cancer cell lines treated with 100 nmol/L MRTX849 for 24 hours or 48 hours. **D,** COX2 mRNA expression in 3LL ΔNRAS and KPAR^G12C^ orthotopic tumors treated for 7 days with 50 mg/kg MRTX849. **E,** COX2-associated inflammatory signature (COX-IS) assessed by qPCR in 3LL ΔNRAS and KPAR^G12C^ orthotopic tumors treated as shown in **D**. **F,** Immunoblot for COX2 in human KRAS^G12C^ lung cancer cell lines treated with MRTX849 for 24 hours. A549 (KRAS^G12S^) cells were used as the negative control. **G,** COX2 expression in RAS-low and RAS-high human lung cancer cell lines from the CCLE database. RPKM, reads per kilobase per million mapped reads. **H,** COX-IS in lung adenocarcinoma samples from TCGA stratified by RAS activity into five different groups, which are associated with specific co-occurring mutations (RAG0, *KRAS* wild-type; RAG1, *KRAS/LKB1*; RAG2, *KRAS*; RAG3, *KRAS/TP53*; RAG4, *KRAS/CDKN2A*). **I,** Immunoblot for COX2 in KPAR^G12C^ cells treated for 2, 3, or 5 days with 100 nmol/L MRTX849. **J,** COX2 mRNA expression in MRTX849 on-treatment and relapsed KPAR^G12C^ tumors. **K,** Kaplan–Meier survival of mice treated with daily oral gavage of 50 mg/kg MRTX849 alone or in combination with 30 mg/kg celecoxib, *n* = 8–20 per group. Analysis of survival curves was carried out using the log-rank (Mantel–Cox) test. Data are represented as mean ± SEM for **A**, **C–E**, and **J**, *n* = 8–9 per group. Groups were compared using unpaired, two-tailed Student *t* test (**A**, **C**–**E**, and **G**) or one-way ANOVA, FDR 0.05 (**H** and **J**). *, *P* < 0.05; **, *P* < 0.01; ***, *P* < 0.001; ****, *P* < 0.0001.

Given the known role of oncogenic KRAS in mediating immune evasion, we next wanted to understand whether tumor-intrinsic COX2 expression was regulated by KRAS signaling. To test this, we inhibited KRAS signaling in several mouse and human KRAS-mutant cancer cell lines. First, treatment of KPAR cells *in vitro* with the MEKi trametinib led to a drastic reduction in COX2 protein expression and loss of PGE2 secretion (Fig. 6A). We validated this in the 3LL ΔNRAS mouse lung cancer cell line (26), which contains a KRAS^G12C^ mutation and has been rendered sensitive to KRAS^G12C^ inhibitors by deletion of oncogenic NRAS, as well as the CT26^G12C^ colorectal cancer cell line (27) and the KPAR^G12C^ cell line (17), which have been engineered to express KRAS^G12C^ (Supplementary Fig. S9A). Treatment with the KRAS^G12C^ inhibitor MRTX849 led to reduced COX2 expression (Fig. 6B) and loss of PGE2 secretion in all three cell lines (Fig. 6C). Inhibition of oncogenic KRAS using the KRAS^G12D^ mutant–specific inhibitor MRTX1133 also resulted in a reduction in COX2 expression in the KPAR^G12D^ cell line (Supplementary Fig. S9B and S9C). Importantly, celecoxib significantly delayed the growth of 3LL ΔNRAS tumors (Supplementary Fig. S9D) and has recently been shown to reduce the growth of CT26 tumors (41). To extend these findings *in vivo*, we carried out gene expression analysis of KP genetically engineered mouse model or KPAR tumors from mice treated with MEKi or the combination of trametinib, everolimus, and linsitinib, respectively, and observed a decrease in the expression of COX2 mRNA (Supplementary Fig. S9E and S9F). MEKi inhibits MAPK signaling in stromal and immune cells as well as tumor cells so we also assessed COX2 expression levels in 3LL ΔNRAS and KPAR^G12C^ tumors from mice treated with MRTX849, as KRAS^G12C^ inhibitors only target tumor cells. KRAS^G12C^ inhibition downregulated COX2 mRNA expression in both 3LL ΔNRAS and KPAR^G12C^ tumors (Fig. 6D). This was accompanied by a decrease in COX2 protein expression (Supplementary Fig. S9G) in KPAR^G12C^ tumors from mice treated with MRTX849. Furthermore, in both tumor models, KRAS^G12C^ inhibition reduced the expression of the COX2-associated inflammatory signature COX-IS (Fig. 6E).

Consistent with our results in mouse cancer cell lines, we also observed significant downregulation of COX2 expression in a panel of KRAS^G12C^ human lung adenocarcinoma cell lines treated *in vitro* with MRTX849 (Fig. 6F). Importantly, such an effect was not observed in KRAS^G12S^ A549 cells, which are insensitive to KRAS^G12C^ inhibition. The KRAS^G12C^ human cell lines differed in their sensitivity to KRAS^G12C^ inhibition (Supplementary Fig. S9H), suggesting that the downregulation of COX2 was not due to drug-induced cytotoxicity. We extended our analysis using expression data from human lung cancer cell lines from the CCLE database, which were classified as either having high or low oncogenic RAS pathway activity based on the 84-gene RAS transcriptional signature (30). We observed significantly increased expression of *PTGS2* in high-RAS pathway activity cell lines (Fig. 6G). Similarly, *PTGS2* expression was increased in KRAS-mutant cell lines (Supplementary Fig. S9I). We also analyzed lung adenocarcinoma expression data from TCGA, which were stratified by hierarchical clustering into five different groups, RAG0 to RAG4, based on expression of the RAS activity transcriptional signature, with RAG0 being the lowest and RAG4 being the highest. COX-IS was significantly increased in all RAS active groups with highest levels occurring in RAG1 and RAG3, which are associated with co-occurring *STK11/LKB1* or *TP53* mutations, respectively (Fig. 6H). Similarly, *PTGS2* mRNA levels were elevated in RAS active tumors as well as KRAS-mutant tumors (Supplementary Fig. S9J). Interestingly, COX-IS was not elevated in KRAS-mutant tumors (Supplementary Fig. S9K), demonstrating the advantage of utilizing a RAS transcriptional signature, rather than simply KRAS mutation status, to capture RAS signaling activity in human lung cancer.

The advent of KRAS^G12C^ inhibitors has transformed the treatment landscape for patients with KRAS^G12C^-mutant lung cancer. However, responses are often short lived, and combination therapies will be required to overcome the development of adaptive resistance. Although oncogenic KRAS pathway reactivation will re-engage proliferative signaling, we postulated that restoration of KRAS-mediated immunosuppression may also contribute to tumor relapse. Interestingly, COX2 expression was restored in KPAR^G12C^ cells *in vitro* after long-term MRTX849 treatment (Fig. 6I) and *in vivo* in KPAR^G12C^ tumors that relapsed after MRTX849 treatment (Fig. 6J). Upregulated expression of multiple KRAS target genes (*Dusp4*, *Dusp5*, and *Dusp6*) in MRTX-relapsed tumors confirmed the reactivation of KRAS pathway signaling *in vivo* (Supplementary Fig. S9L). Importantly, the combination of MRTX849 and celecoxib delayed tumor relapse and significantly improved the survival of KPAR^G12C^ tumor–bearing mice compared with MRTX849 alone (Fig. 6K).

In summary, these data suggest that oncogenic KRAS is a major driver of COX2/PGE2 signaling in mouse and human lung cancer and may therefore contribute to ICB resistance in KRAS-mutant lung adenocarcinoma and tumor relapse in patients treated with KRAS^G12C^ inhibitors, which can be overcome by COX2/PGE2 pathway inhibitors.

## Discussion

A subset of patients with KRAS-mutant lung cancer has greatly benefited from the advent of ICB therapy. However, the majority of patients still do not respond. Combination strategies are urgently required to broaden the efficacy of immunotherapy. Accumulating evidence suggests that tumor-intrinsic oncogenic signaling can dampen antitumor immune responses (30), which could be therapeutically exploited to overcome immunotherapy resistance in specific tumor subtypes, but little progress has been made to date in attempts to combine immunotherapies with therapies targeting oncogenic signaling pathways. Here, we carried out *in vivo* CRISPR screening using a novel immunogenic model of KRAS-mutant lung adenocarcinoma (17) to uncover multiple mechanisms by which oncogenic KRAS drives immune evasion, with a view to inform the development of optimal combination strategies for targeting KRAS-mutant cancers that impact both oncogenic signaling and immune evasion.

We demonstrated that tumor-intrinsic IFNγ signaling is critical for antitumor immunity. Indeed, numerous CRISPR screens have demonstrated that defects in IFNγ signaling result in resistance to immunotherapy (21, 43) and mutations in *JAK1* and *JAK2* have been associated with acquired resistance to ICB (44). Given the importance of tumor-intrinsic IFNγ signaling in sensitizing KPAR tumors to antitumor immune responses, the ability of oncogenic KRAS to suppress IFN pathway signaling may represent a major mechanism of immune evasion in KRAS-mutant lung adenocarcinoma. These results are consistent with our previous findings that KRAS^G12C^ inhibitors can restore tumor-intrinsic IFN signaling in multiple preclinical models of lung cancer (15). Paradoxically, tumor-intrinsic IFNγ signaling has also been shown to impede antitumor immunity (45), including in a CRISPR screen using an orthotopic KRAS^G12D^ p53^−/−^ lung cancer model similar to the one used in this study (25). Although such opposing effects in similar models are surprising, chronic tumor-intrinsic IFNγ signaling can drive resistance to ICB because of the upregulation of immune checkpoint ligands (46). Understanding the contexts in which tumor-intrinsic IFNγ signaling promotes or impedes antitumor immunity will be important when assessing combination strategies targeting this pathway.

We also identified KRAS-driven expression of COX2 and secretion of PGE2 as a major mechanism of immune evasion, which suppresses both innate and adaptive antitumor immune responses. This is consistent with the role of tumor-intrinsic COX2 in driving immune evasion in melanoma and colorectal cancer (37). The ability of our CRISPR screen to elucidate the function of a secreted molecule can be explained by a recent barcoded CRISPR screen that demonstrated that orthotopic KP tumors grow as clonal lesions (25), suggesting that the local secretion of a particular molecule by neighboring tumor cells that do not contain the same gene deletion is unlikely to be a major problem.

COX2-derived PGE2 is a pleiotropic molecule that has been shown to act on many cell types including CD8^+^ T cells, DCs, and NK cells (47), although single-cell sequencing studies have revealed EP2 and EP4 receptors to be highly expressed specifically on tumor-infiltrating myeloid cells (48). Indeed, we observed extensive changes in the TME of COX2-deficient tumors including proinflammatory polarization of myeloid cells and concomitant infiltration of highly activated CD8^+^ T cells and NK cells. Interestingly, NK cell depletion was best able to restore the growth of COX2-deficient tumors. Although NK cells play a major role in the control of orthotopic lung cancer models, we also observed a reduced growth of COX2-deficient subcutaneous tumors as early as 7 days, before the onset of an adaptive immune response. This is consistent with the observation that PGE2 directly suppresses the survival of NK cells (49), which are required to drive remodeling of the TME, including proinflammatory polarization of macrophages and activation of CD8^+^ T cells, and promote immune control in COX2-deficient melanoma tumors (31). The dramatic reduction of survival upon NK-cell depletion also suggests a role for direct tumor cell killing, which we have previously shown to be mediated by Ab-dependent cellular cytotoxicity in the KPAR model (50). Furthermore, we observed that the effects of tumor-derived PGE2 on myeloid cells are consistent with a recent study that demonstrated the immunosuppressive role of PGE2-producing lung fibroblasts, which promote breast cancer metastasis (40). The role of COX2-expressing lung fibroblasts within primary lung tumors remains unclear; however, our results suggest that the majority of PGE2 secretion within the TME is derived from tumor cells, as tumor-specific genetic deletion of COX2 was sufficient to drive immune-mediated tumor eradication. Unlike studies in melanoma (37), we did not observe increased CD103^+^ DC recruitment in COX2-deficient tumors, reflecting the context-specific effects of prostaglandin signaling in different tumor types. CD103^+^ DCs have a unique ability to prime CD8^+^ T cells, and the increased expression of costimulatory molecule expression on CD103^+^ DCs in *Ptgs2*^−/−^ tumors may explain the enhanced T-cell activation observed. A recent study utilizing the syngeneic 3LL/LLC Lewis lung cancer model described a role for Tregs in mediating the immunosuppressive effects of the COX2/PGE2 signaling axis in subcutaneous tumors (48), which we did not observe in our experimental model system. This may be explained by differences in antitumor immune responses observed in orthotopic versus subcutaneous tumors. Indeed, multiple transplantable lung cancer models have been shown to have abundant infiltration of Tregs in subcutaneous tumors compared with orthotopic lung tumors (17, 51). COX2-deficient tumors exhibited increased abundance of Tregs, which is consistent with murine experiments demonstrating that CD8^+^ T cells can drive Treg infiltration into the TME (52).

Our data demonstrate the therapeutic benefits of genetic ablation or pharmacologic inhibition of COX2 in a preclinical model of KRAS-mutant lung cancer, especially in combination with immunotherapy. Furthermore, the ability of the COX-IS to independently predict the outcome after ICB suggests a role for COX2/PGE2 signaling in hindering responses to ICB in patients with lung adenocarcinoma, supporting previous findings in other cancer types (31). Given that the expression of the COX-IS was strongly driven by oncogenic KRAS signaling in both mouse and human lung cancer, our studies suggest that inhibiting the COX2/PGE2 axis is a promising therapeutic strategy in KRAS-mutant non–small cell lung cancer and may broaden the efficacy of ICB for these patients. Interestingly, our analysis of COX-IS expression in TCGA lung adenocarcinoma cohort demonstrated increased activity of the COX2/PGE2 axis in RAG1 patients, which are associated with STK11/LKB1 co-occurring mutations. These patients respond poorly to ICB (53) and therefore may particularly benefit from this combination therapy.

Indeed, a number of clinical trials are currently testing the combination of celecoxib with ICB (NCT03026140 and NCT03864575). However, these trials do not specifically enroll patients with KRAS-mutant lung adenocarcinoma, which our data suggest would most benefit from such a combination. The potential benefit of this combination is supported by a recent retrospective analysis showing improved survival of patients with lung cancer that were concurrently treated with COX inhibitors while receiving immunotherapy (54). Furthermore, we show using a novel drug that dual inhibition of the PGE2 receptors EP2 and EP4 has similar therapeutic benefits to COX2 inhibition and shows superior synergy when combined with ICB. This supports other work in preclinical models that have demonstrated the benefits of combining EP2 and EP4 antagonists with ICB (40, 41, 55). EP2 and EP4 inhibition, therefore, has the potential to enhance the efficacy of immunotherapy in the clinic while possibly avoiding the toxicities associated with celecoxib treatment, including those associated with inhibition of other targets of PGE2, including EP1 and EP3 receptors.

The ability of KRAS^G12C^ inhibition to suppress the COX2/PGE2 signaling axis *in vivo* may, in part, explain the synergy observed in combination with ICB in preclinical models (15, 17, 18, 27). However, given the apparent poor tolerability of the combination of KRAS^G12C^ inhibition by sotorasib and PDL1-targeted ICB observed in the clinic (19), it may be more feasible to target KRAS-driven immune evasion mechanisms such as COX2. The benefits of KRAS^G12C^ inhibition in the clinic are also seriously confounded by the rapid emergence of acquired resistance, which can be driven by many different oncogenic mutations within the RAS signaling pathway (11). Clinical trials such as CodeBreaK 101 are attempting to overcome this by combining KRAS^G12C^ inhibitors with other targeted therapies such as receptor tyrosine kinase inhibitors; however, given the genetic complexity underlying resistance, the feasibility of this approach remains unclear. Instead, targeting KRAS-driven immune suppression may prove more successful. Our work shows that long-term KRAS^G12C^ inhibition results in restoration of the COX2/PGE2 axis, which may contribute to tumor relapse. Furthermore, our data suggest that the combination of KRAS^G12C^ inhibitory drugs and COX2 or EP2 to EP4 prostaglandin receptor inhibition may be successful in the treatment of immune hot lung cancer, and it might be speculated that it could possibly avoid the toxicities reported for sotorasib and PDL1 blockade.

## Supplementary Material

Supplementary Figure 1 In vivo screen identifies mediators of immune resistance and sensitivity

Supplementary Figure 2 Supplementary Figure 2. KRAS-driven inhibition of tumor-intrinsic IFN signaling promotes immune evasion

Supplementary Figure 3 Supplementary Figure 3. Oncogenic KRAS inhibits tumor-intrinsic IFN responses via Myc

Supplementary Figure 4 Supplementary Figure 4. COX-2 deficient tumors are sensitized to anti-tumor immunity

Supplementary Figure 5 Supplementary Figure 5. Flow cytometry gating strategies

Supplementary Figure 6 Supplementary Figure 6. Tumor-intrinsic COX-2 remodels the lung tumor microenvironment

Supplementary Figure 7 Supplementary Figure 7. Genetic loss of COX-2 signaling synergizes with ICB

Supplementary Figure 8 Supplementary Figure 8. COX-2/PGE2 pathway inhibition remodels the TME and enhances the efficacy of ICB

Supplementary Figure 9 Supplementary Figure 9. Oncogenic KRAS drives tumor-intrinsic expression of COX-2 in LUAD

Supplementary Table 1 Supplementary Table S1. CRISPR-Cas9 screen results

Supplementary Table 2 Supplementary Table S2. Antibodies used for FACS analysis of mouse tumors

Supplementary Table 3 Supplementary Table S3. sgRNA sequences for CRISPR-Cas9 gene knockout
