# Supplementary material for: CRISPR–Cas9 Screening Identifies KRAS-Induced COX2 as a Driver of Immunotherapy Resistance in Lung Cancer
Source: Cancer Res. 2024 Apr 18;84(14):2231–46. doi: 10.1158/0008-5472.CAN-23-2627 (PMC11247323; doi:10.1158/0008-5472.CAN-23-2627)
Supplement: Supplementary Figure 1 — In vivo screen identifies mediators of immune resistance and sensitivity [file can-23-2627_supplementary_figure_1_suppsf1.pdf]

Supp Figure 1

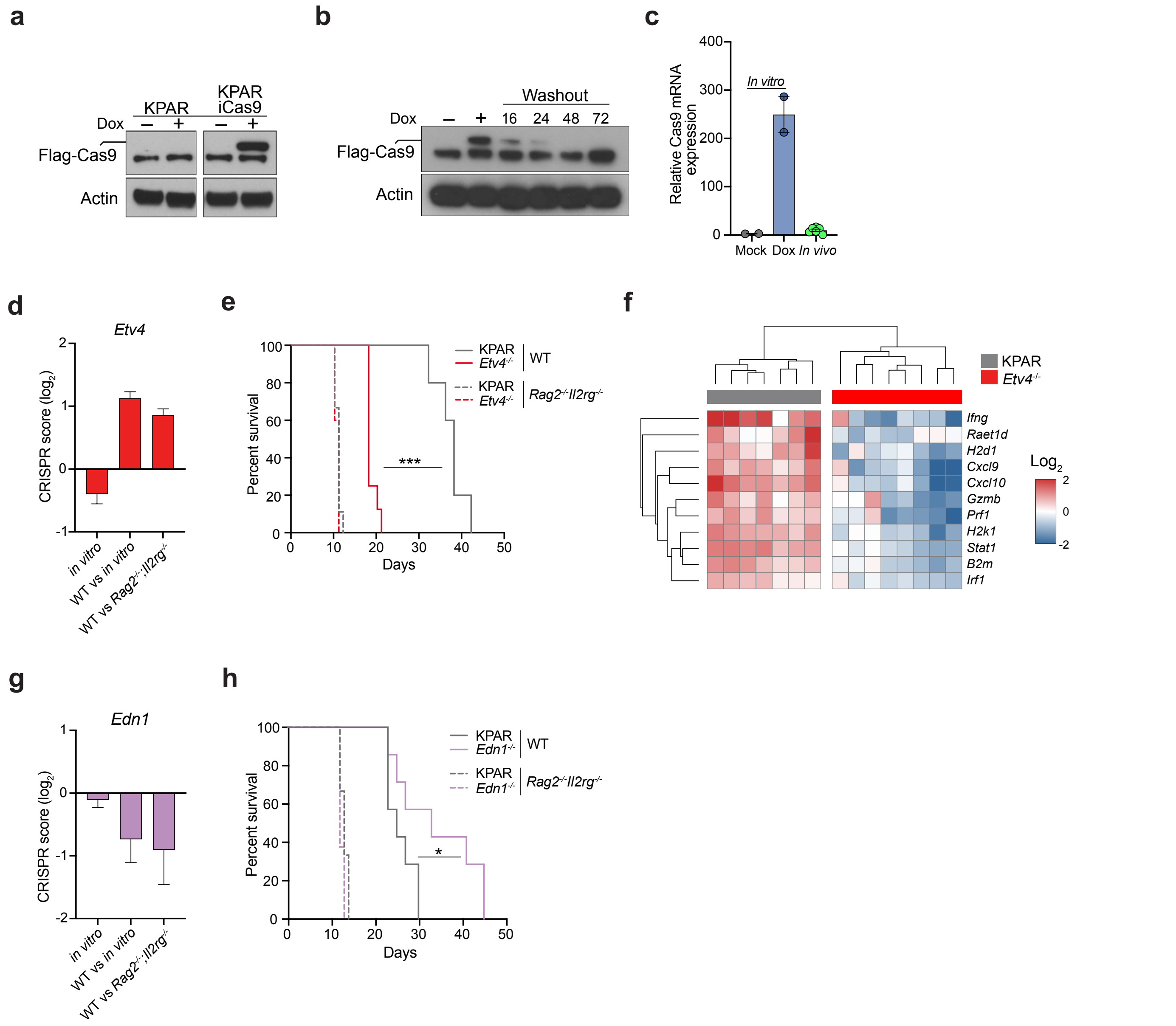

Supplementary Figure 1. ***In vivo*** **screen identifies mediators of immune resistance and sensitivity**
(A) Immunoblot for Flag-Cas9 in KPAR iCas9 cells treated for 24h with 1μM doxycycline. Parental KPAR cells were used as a control.
(B) Immunoblot for Flag-Cas9 in KPAR iCas9 cells treated as in (A) followed by washout at stated time points.
(C) mRNA expression by qPCR of Cas9 in KPAR iCas9 cells treated in vitro with 1μM doxycycline or KPAR iCas9 subcutaneous tumours.
(D) Enrichment of sgRNAs targeting *Etv4* in WT versus *Rag2*$^{-/-}$;*Il2rg*$^{-/-}$ mice.
(E) Kaplan-Meier survival of immune-competent or *Rag2*$^{-/-}$; *Il2rg*$^{-/-}$ mice following orthotopic transplantation with KPAR cells or *Etv4*$^{-/-}$ cells, n=5-9 per group.
(F) Heatmap showing hierarchical clustering of KPAR and *Etv4*$^{-/-}$ tumours based on mRNA expression of immune-related genes assessed by qPCR, n=7-8 per group.
(G) Depletion of sgRNAs targeting *Edn1* in WT versus *Rag2*$^{-/-}$;*Il2rg*$^{-/-}$ mice.
(H) Kaplan-Meier survival of immune-competent or *Rag2*$^{-/-}$; *Il2rg*$^{-/-}$ mice following orthotopic transplantation with KPAR cells or *Edn1*$^{-/-}$ cells, n=6-8 per group. Data are mean ± SEM for (C-D and G). For (E and H), analysis of survival curves was carried out using log-rank (Mantel-Cox) test; * P<0.05, *** P<0.001.
