# Supplementary material for: CRISPR–Cas9 Screening Identifies KRAS-Induced COX2 as a Driver of Immunotherapy Resistance in Lung Cancer
Source: Cancer Res. 2024 Apr 18;84(14):2231–46. doi: 10.1158/0008-5472.CAN-23-2627 (PMC11247323; doi:10.1158/0008-5472.CAN-23-2627)
Supplement: Supplementary Figure 2 — KRAS-driven inhibition of tumor-intrinsic IFN signaling promotes immune evasion [file can-23-2627_supplementary_figure_2_suppsf2.pdf]

Supp Figure 2

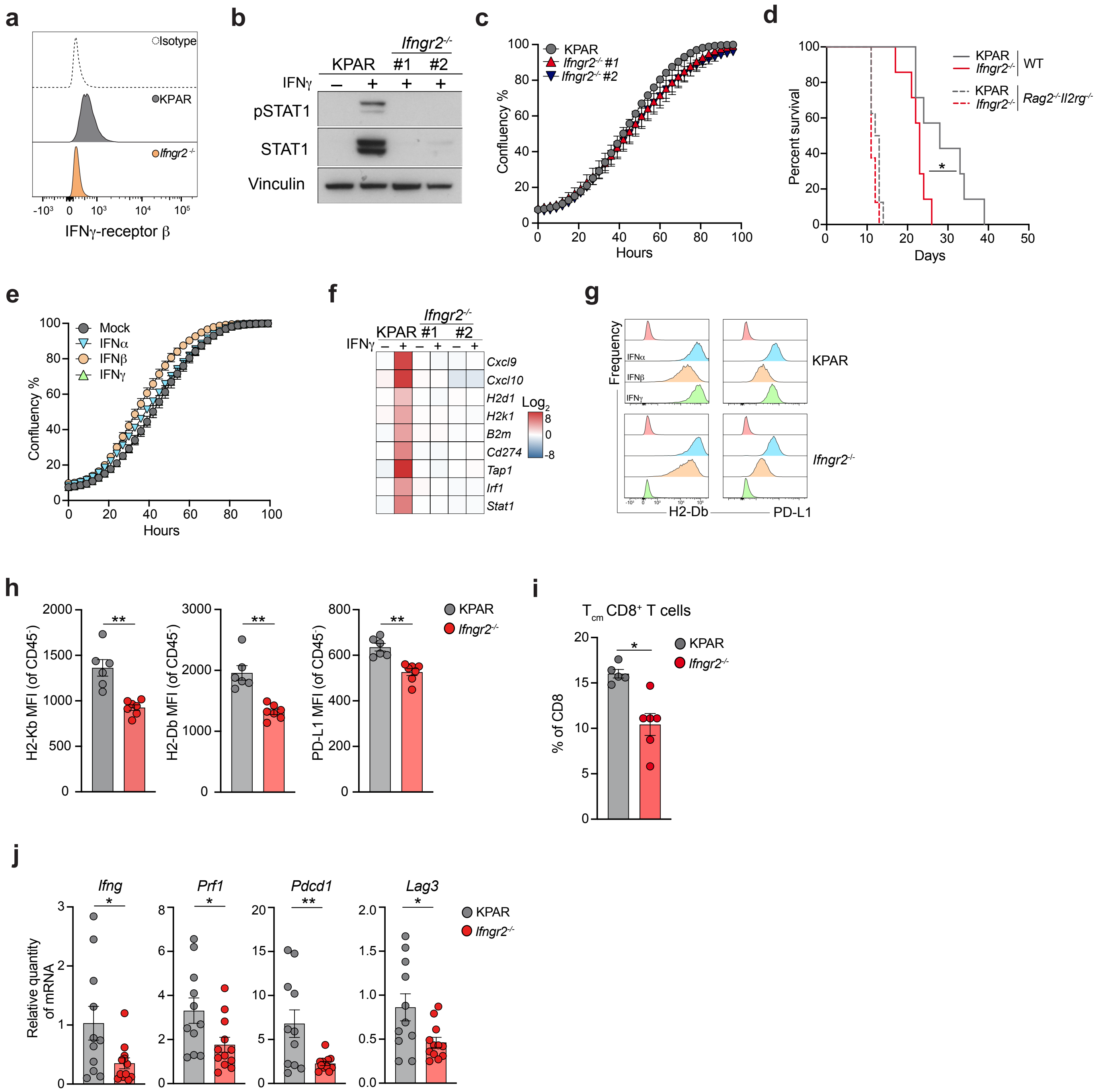

Supplementary Figure 2. KRAS-driven inhibition of tumour-intrinsic IFN signalling promotes immune evasion

(A) Flow cytometry analysis showing surface expression of IFN-γ receptor subunit β on KPAR cells and *Ifngr2*$^{-/-}$ cells.
(B) Immunoblot for pSTAT1 and STAT1 in KPAR cells and *Ifngr2*$^{-/-}$ cells treated for 24h with 100ng/ml IFNγ.
(C) Incucyte analysis showing growth rate of KPAR cells and *Ifngr2*$^{-/-}$ cells *in vitro*.
(D) Kaplan-Meier survival of immune-competent or *Rag2*$^{-/-}$*;Il2rg*$^{-/-}$ mice following orthotopic transplantation with KPAR cells or *Ifngr2*$^{-/-}$ cells, n=5-10 per group. Analysis of survival curves was carried out using log-rank (Mantel-Cox) test; * P<0.05.
(E) Incucyte analysis showing growth of KPAR cells in the presence of 200ng/ml IFNα, 200ng/ml IFNβ or 100ng/ml IFNγ.
(F) Heatmap showing mRNA expression of IFN-response genes in KPAR cells and *Ifngr2*$^{-/-}$ cells treated with 100ng/ml IFNγ.
(G) Flow cytometry analysis showing surface expression of H2-Db (left) and PD-L1 (right) on KPAR cells and Ifngr2$^{-/-}$ cells treated for 24h with either 200ng/ml IFNα, 200ng/ml IFNβ or 100ng/ml IFNγ.
(H) Flow cytometry analysis showing surface expression (mean fluorescence intensity) of H2-Kb, H2-Db and PD-L1 on CD45^{-} cells in KPAR and *Ifngr2*$^{-/-}$ tumours.
(I) Frequency of tumour-infiltrating central memory (T$_{cm}$ – CD62L^{+} CD44^{+}) CD8^{+} T cells.
(J) mRNA expression by qPCR of immune-related genes. Data are mean ± SEM for (H-J), n=5-12 per group. Groups were compared using un-paired, two-tailed Student's t-test; * P<0.05, ** P<0.01.
