# Supplementary material for: CRISPR–Cas9 Screening Identifies KRAS-Induced COX2 as a Driver of Immunotherapy Resistance in Lung Cancer
Source: Cancer Res. 2024 Apr 18;84(14):2231–46. doi: 10.1158/0008-5472.CAN-23-2627 (PMC11247323; doi:10.1158/0008-5472.CAN-23-2627)
Supplement: Supplementary Figure 3 — Oncogenic KRAS inhibits tumor-intrinsic IFN responses via Myc [file can-23-2627_supplementary_figure_3_suppsf3.pdf]

Supp Figure 3

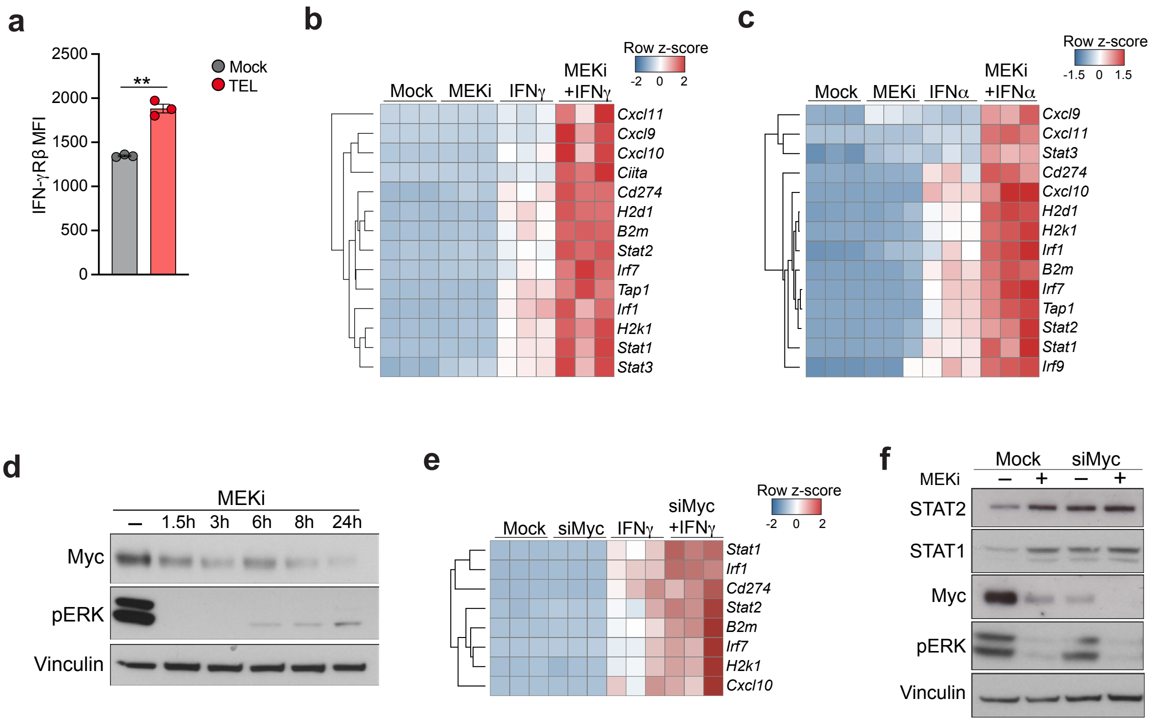

Supplementary Figure 3. Oncogenic KRAS inhibits tumour-intrinsic IFN responses via Myc
(A) Surface expression (mean fluoresence intensity) of the IFNγ-receptor β chain on KPAR cells treated with 10nM trametinib, 1μM linsitinib and 40nM everolimus (TEL) for 24h. Data are mean ± SEM, n=3 per group. Groups were compared using unpaired, two-tailed Student's t-test; ** P<0.01.
(B-C) Heatmap showing expression of IFN-response genes by qPCR in KPAR cells treated for 24h with 100ng/ml recombinant IFNγ (B) or 200ng/ml 200ng/ml IFNα (C) in combination with 10nM trametinib (MEKi).
(D) Immunoblot for Myc in KPAR cells treated at indicated time points with 10nM trametinib.
(E) Heatmap showing expression of IFN-response genes in KPAR cells after siRNA-mediated knockdown of Myc and treatment with 100ng/ml IFNγ.
(F) Immunoblot for STAT1 and STAT2 in KPAR cells after siRNA-mediated knockdown of Myc and treatment with 10nM trametinib for 24h.
