# Supplementary material for: CRISPR–Cas9 Screening Identifies KRAS-Induced COX2 as a Driver of Immunotherapy Resistance in Lung Cancer
Source: Cancer Res. 2024 Apr 18;84(14):2231–46. doi: 10.1158/0008-5472.CAN-23-2627 (PMC11247323; doi:10.1158/0008-5472.CAN-23-2627)
Supplement: Supplementary Figure 4 — COX-2 deficient tumors are sensitized to anti-tumor immunity [file can-23-2627_supplementary_figure_4_suppsf4.pdf]

Supp Figure 4

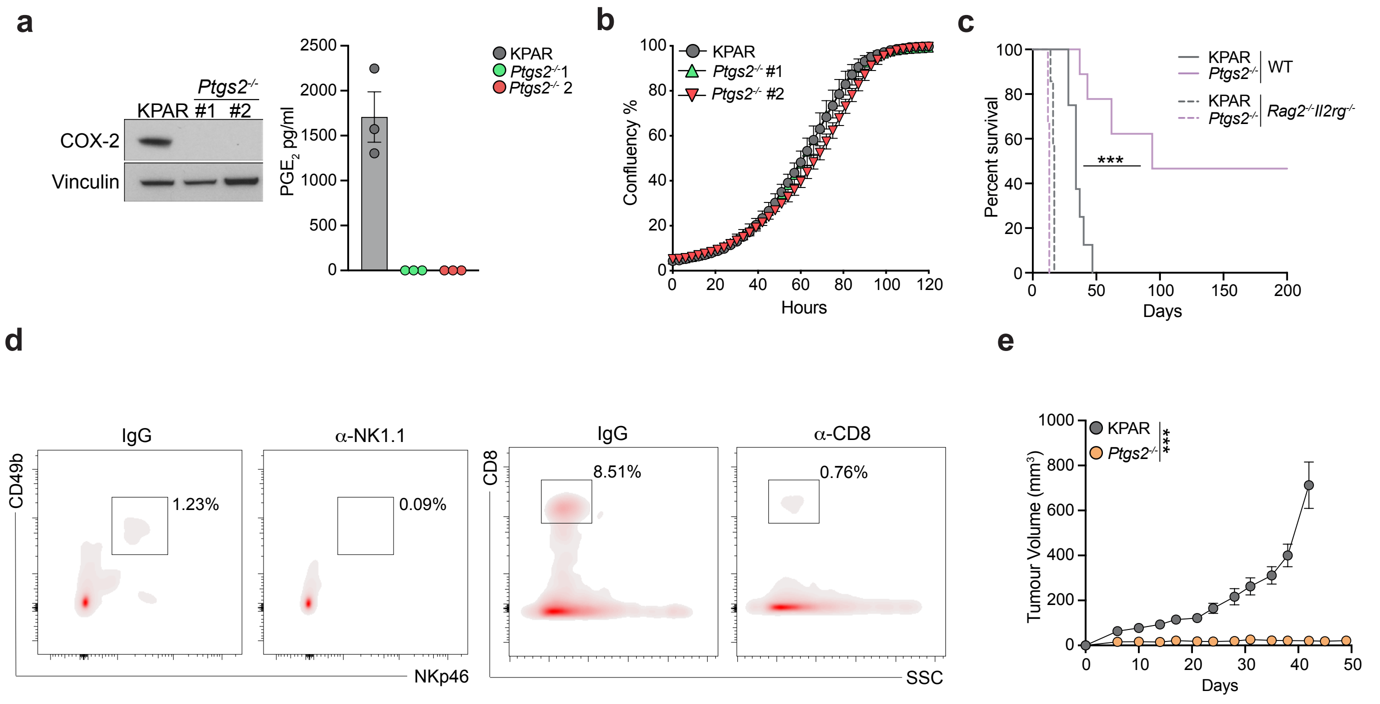

Supplementary Figure 4. COX-2 deficient tumors are sensitised to anti-tumour immunity

(A) Immunoblot for COX-2 (left) and ELISA analysis for PGE_2 concentration (right) in KPAR cells and *Ptgs2*$^{-/-}$ cells.

(B) Incucyte analysis showing growth rate of KPAR cells and *Ptgs2*$^{-/-}$ cells *in vitro*.

(C) Kaplan-Meier survival of immune-competent or *Rag2*$^{-/-}$; *Il2rg*$^{-/-}$ mice following orthotopic transplantation with KPAR cells or *Ptgs2*$^{-/-}$ cells (clone 2), n=6-9 per group. Analysis of survival curves was carried out using log-rank (Mantel-Cox) test; *** P<0.001.

(D) Representative flow cytometry plots showing frequency of NK cells (left) or $CD8^+$ T cells (right) in spleens from mice 1 week after treatment with depleting antibodies.

(E) Growth of subcutaneous KPAR and *Ptgs2*$^{-/-}$ tumours in immune-competent mice, n=10 per group. Groups were compared by two-way ANOVA; *** P<0.001.
