# Supplementary material for: CRISPR–Cas9 Screening Identifies KRAS-Induced COX2 as a Driver of Immunotherapy Resistance in Lung Cancer
Source: Cancer Res. 2024 Apr 18;84(14):2231–46. doi: 10.1158/0008-5472.CAN-23-2627 (PMC11247323; doi:10.1158/0008-5472.CAN-23-2627)
Supplement: Supplementary Figure 5 — Flow cytometry gating strategies [file can-23-2627_supplementary_figure_5_suppsf5.pdf]

Supp Figure 5

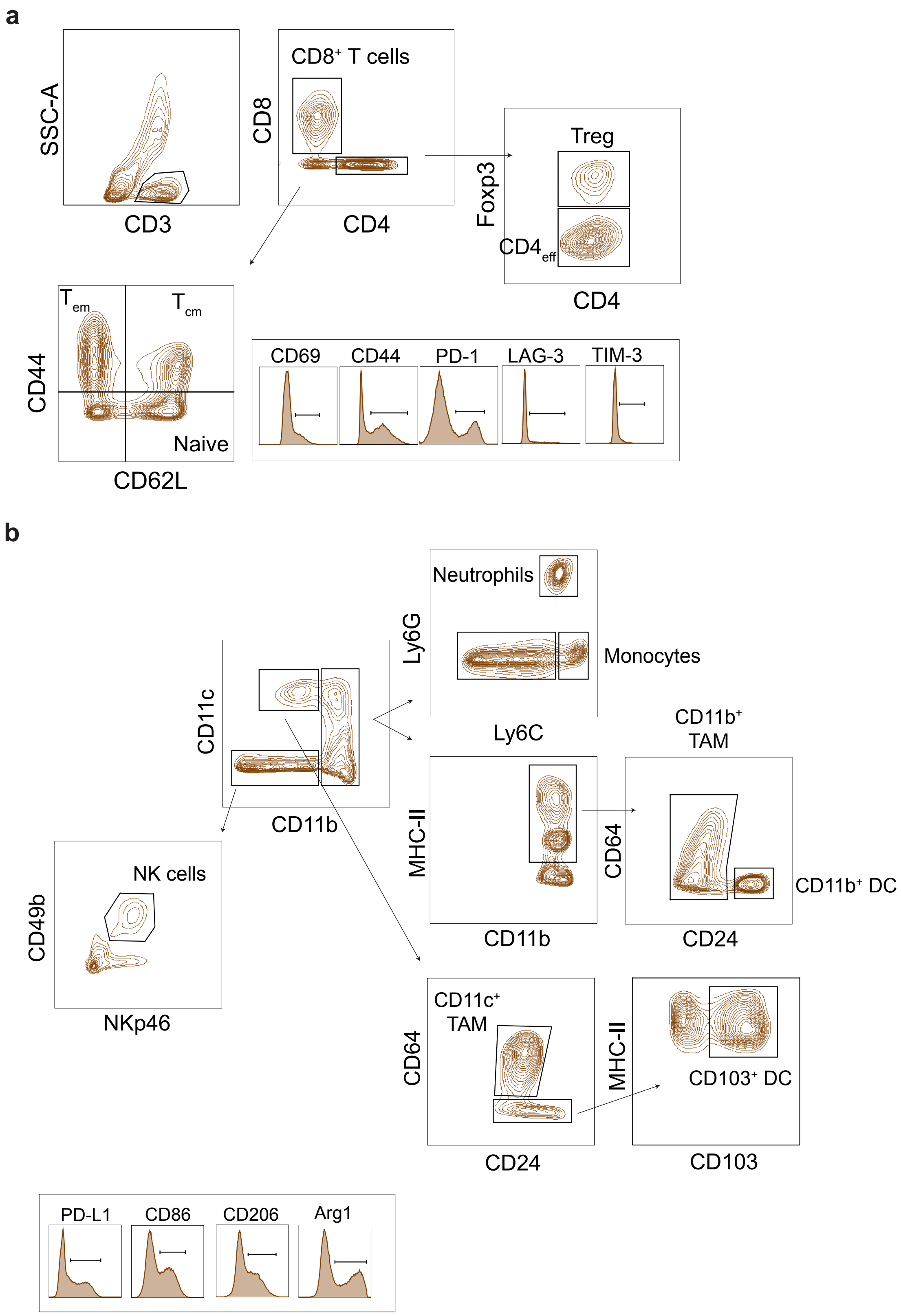

Supplementary Figure 5. Flow cytometry gating strategies
(A-B) Representative gating for the identification of different T cell subsets (A) and myeloid cells (B) and expression of phenotypic markers.
