# Supplementary material for: CRISPR–Cas9 Screening Identifies KRAS-Induced COX2 as a Driver of Immunotherapy Resistance in Lung Cancer
Source: Cancer Res. 2024 Apr 18;84(14):2231–46. doi: 10.1158/0008-5472.CAN-23-2627 (PMC11247323; doi:10.1158/0008-5472.CAN-23-2627)
Supplement: Supplementary Figure 6 — Tumor-intrinsic COX-2 remodels the lung tumor microenvironment [file can-23-2627_supplementary_figure_6_suppsf6.pdf]

Supp Figure 6

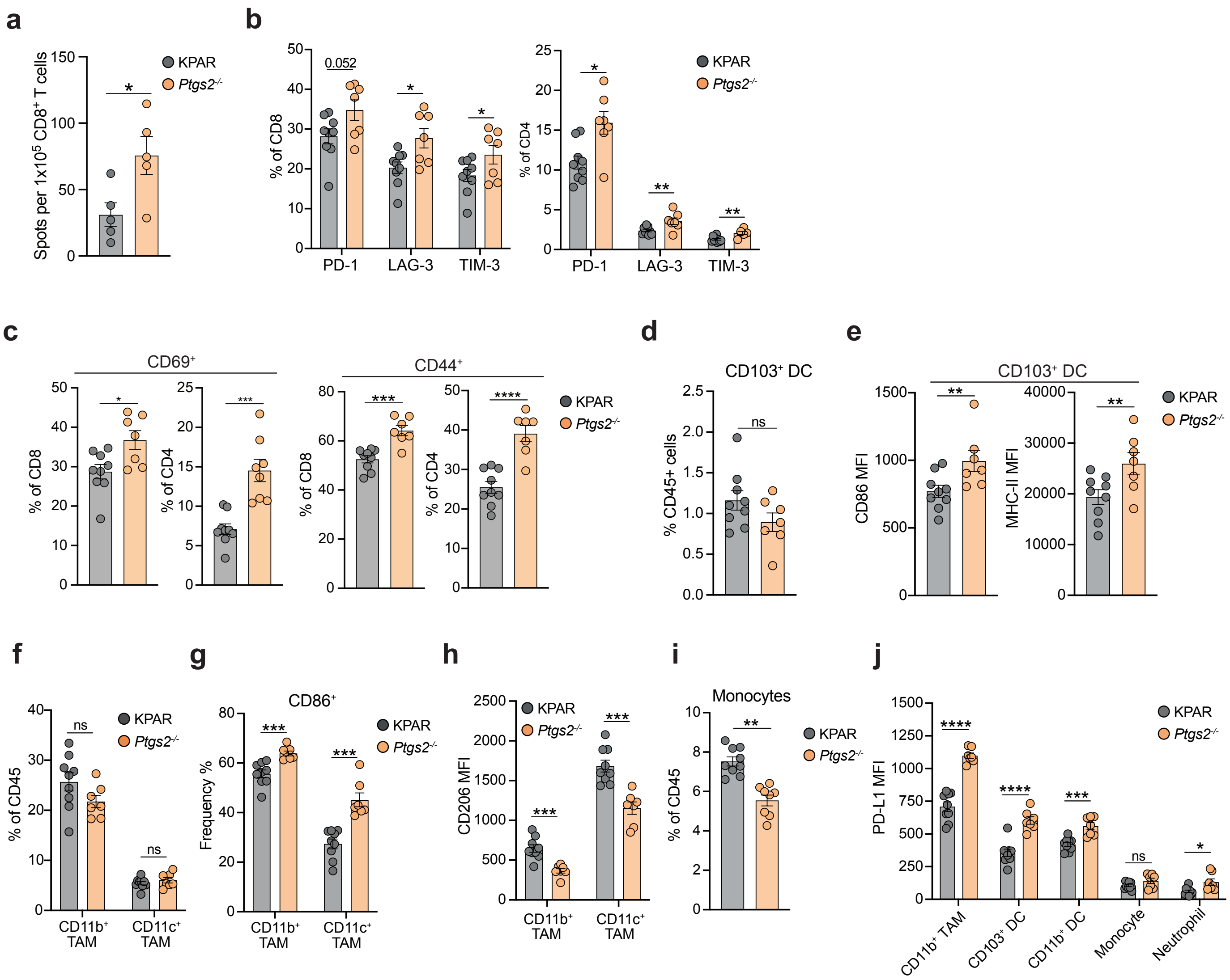

Supplementary Figure 6. Tumour-intrinsic COX-2 remodels the lung tumour microenvironment
(A) IFNγ ELISPOT analysis of CD8⁺ T cells isolated from KPAR and *Ptgs2*$^{-/-}$ tumor-bearing mice pulsed with eMLV *env* peptide.
(B) Frequency of PD-1⁺, LAG-3⁺ and TIM-3⁺ CD8⁺ (left) and CD4⁺ (right) T cells in KPAR and *Ptgs2*$^{-/-}$ tumours.
(C) Percentage of CD69⁺ (left) and CD44⁺ (right) T cells.
(D-E) Frequency of tumour-infiltrating CD103⁺ DCs (D) and surface expression (mean fluoresence intensity) of CD86 and MHC-II on CD103⁺ DCs (E).
(F-G) Frequency of tumour-infiltrating CD11b⁺ and CD11c⁺ TAMs (F) and percentage of CD86⁺ TAMs (G).
(H) Surface expression (mean fluoresence intensity) of CD206 on TAMs.
(I) Frequency of Ly6C⁺ monocytes.
(J) Surface expression (mean fluoresence intensity) of PD-L1 on myeloid cell populations.
Data are mean ± SEM, n=5=10 per group. Samples were analysed using unpaired, two-tailed Student's t-test; ns, not significant, * P<0.05, ** P<0.01, *** P<0.001, **** P<0.0001.
