# Supplementary material for: CRISPR–Cas9 Screening Identifies KRAS-Induced COX2 as a Driver of Immunotherapy Resistance in Lung Cancer
Source: Cancer Res. 2024 Apr 18;84(14):2231–46. doi: 10.1158/0008-5472.CAN-23-2627 (PMC11247323; doi:10.1158/0008-5472.CAN-23-2627)
Supplement: Supplementary Figure 7 — Genetic loss of COX-2 signaling synergizes with ICB [file can-23-2627_supplementary_figure_7_suppsf7.pdf]

Supp Figure 7

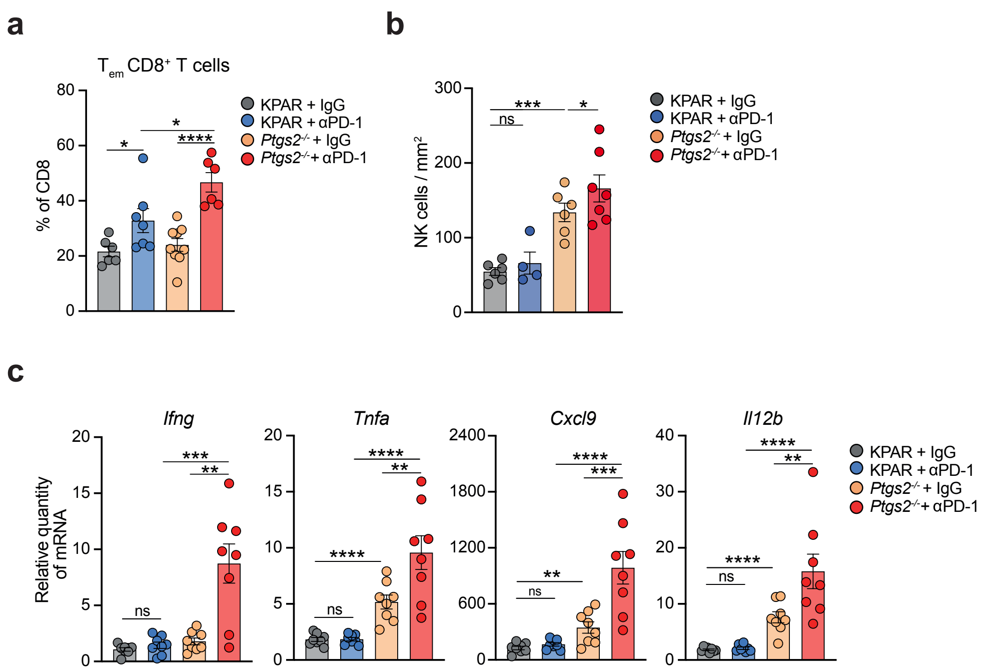

Supplementary Figure 7. Genetic loss of COX-2 signalling synergises with ICB
(A) Percentage of effector memory CD8+ T cells in KPAR or *Ptgs2*$^{-/-}$ tumours on day 7 after treatment with anti-PD-1 or corresponding isotype control (IgG).
(B) Quantification of NK cells by immunohistochemistry in KPAR or *Ptgs2*$^{-/-}$ orthotopic tumours on day 7 after treatment with anti-PD-1 or corresponding isotype control (IgG).
(C) mRNA expression by qPCR of anti-tumour immunity genes in KPAR or *Ptgs2*$^{-/-}$ tumours treated as in (A).
Data are mean ± SEM, n=4-8 per group. Statistics were calculated using one-way ANOVA, FDR 0.05; ns, not significant, * P<0.05, ** P<0.01, *** P<0.001, **** P<0.0001.
