# Supplementary material for: CRISPR–Cas9 Screening Identifies KRAS-Induced COX2 as a Driver of Immunotherapy Resistance in Lung Cancer
Source: Cancer Res. 2024 Apr 18;84(14):2231–46. doi: 10.1158/0008-5472.CAN-23-2627 (PMC11247323; doi:10.1158/0008-5472.CAN-23-2627)
Supplement: Supplementary Figure 8 — COX-2/PGE2 pathway inhibition remodels the TME and enhances the efficacy of ICB [file can-23-2627_supplementary_figure_8_suppsf8.pdf]

Supp Figure 8

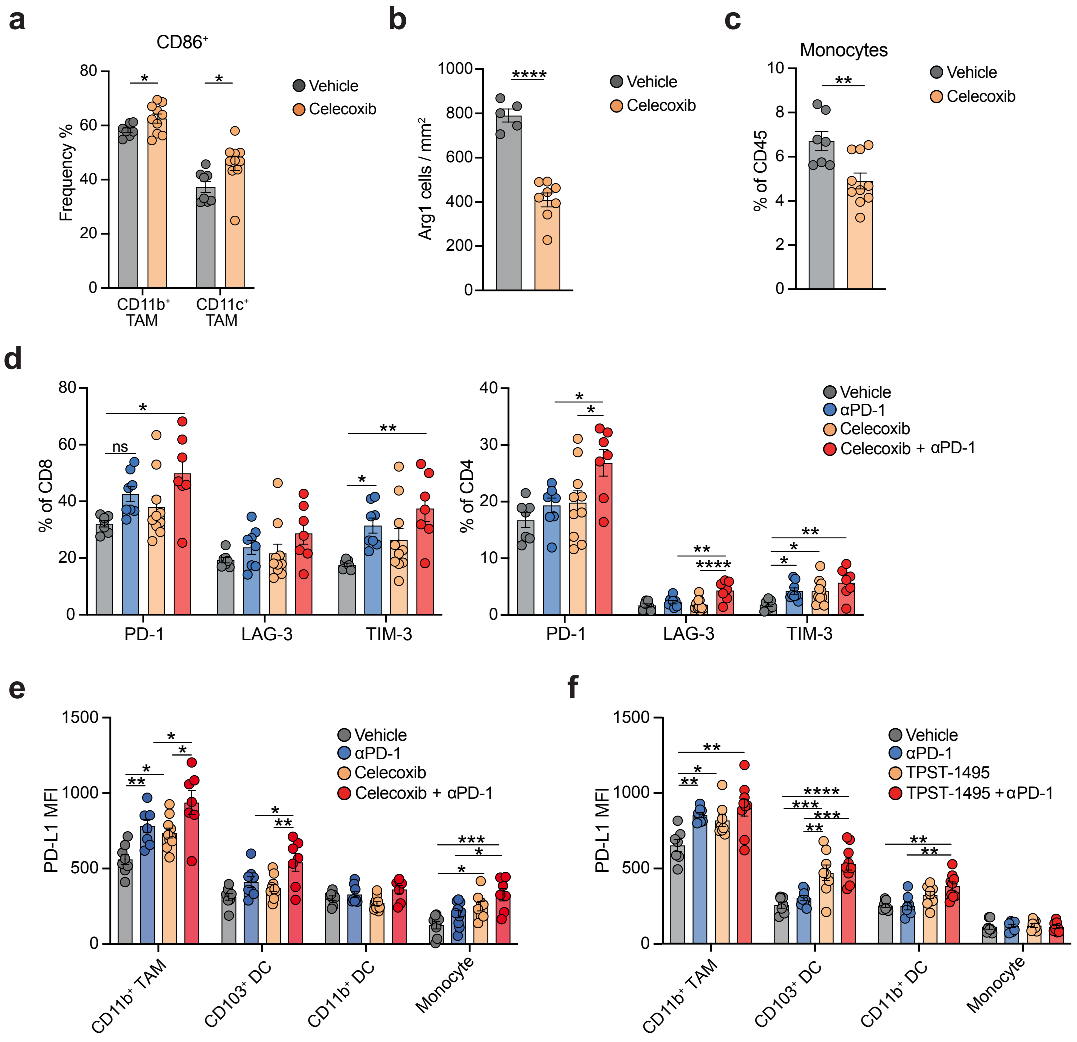

Supplementary Figure 8. COX-2/PGE2 pathway inhibition remodels the TME and enhances the efficacy of ICB

(A) Percentage of $CD86^+$ TAMs in KPAR orthotopic tumours treated for 7d with 30mg/kg celecoxib.

(B) Quantification of $Arg1^+$ cells by immunohistochemistry in KPAR orthotopic tumours treated as in (A).

(C) Frequency of $Ly6C^+$ monocytes in KPAR tumours treated as in (A).

(D) Frequency of $PD\text{-}1^+$, $LAG\text{-}3^+$ and $TIM\text{-}3^+$ $CD8^+$ (left) and $CD4^+$ (right) T cells in KPAR tumours treated for 7d with celecoxib and/or anti-PD-1.

(E-F) Surface expression (mean fluoresence intensity) of PD-L1 on myeloid cell populations in KPAR tumours treated for 7d with anti-PD-1 and/or celecoxib (E) or 100mg/kg TPST-1495 (F).

Data are mean ± SEM, n=5-10 per group. Groups were compared using unpaired, two-tailed Student's t-test (A-C) or one-way ANOVA, FDR 0.05 (D-F); ns, not significant, * $P<0.05$, ** $P<0.01$, *** $P<0.001$, **** $P<0.0001$.
