# Supplementary material for: CRISPR–Cas9 Screening Identifies KRAS-Induced COX2 as a Driver of Immunotherapy Resistance in Lung Cancer
Source: Cancer Res. 2024 Apr 18;84(14):2231–46. doi: 10.1158/0008-5472.CAN-23-2627 (PMC11247323; doi:10.1158/0008-5472.CAN-23-2627)
Supplement: Supplementary Figure 9 — Oncogenic KRAS drives tumor-intrinsic expression of COX-2 in LUAD [file can-23-2627_supplementary_figure_9_suppsf9.pdf]

Supp Figure 9

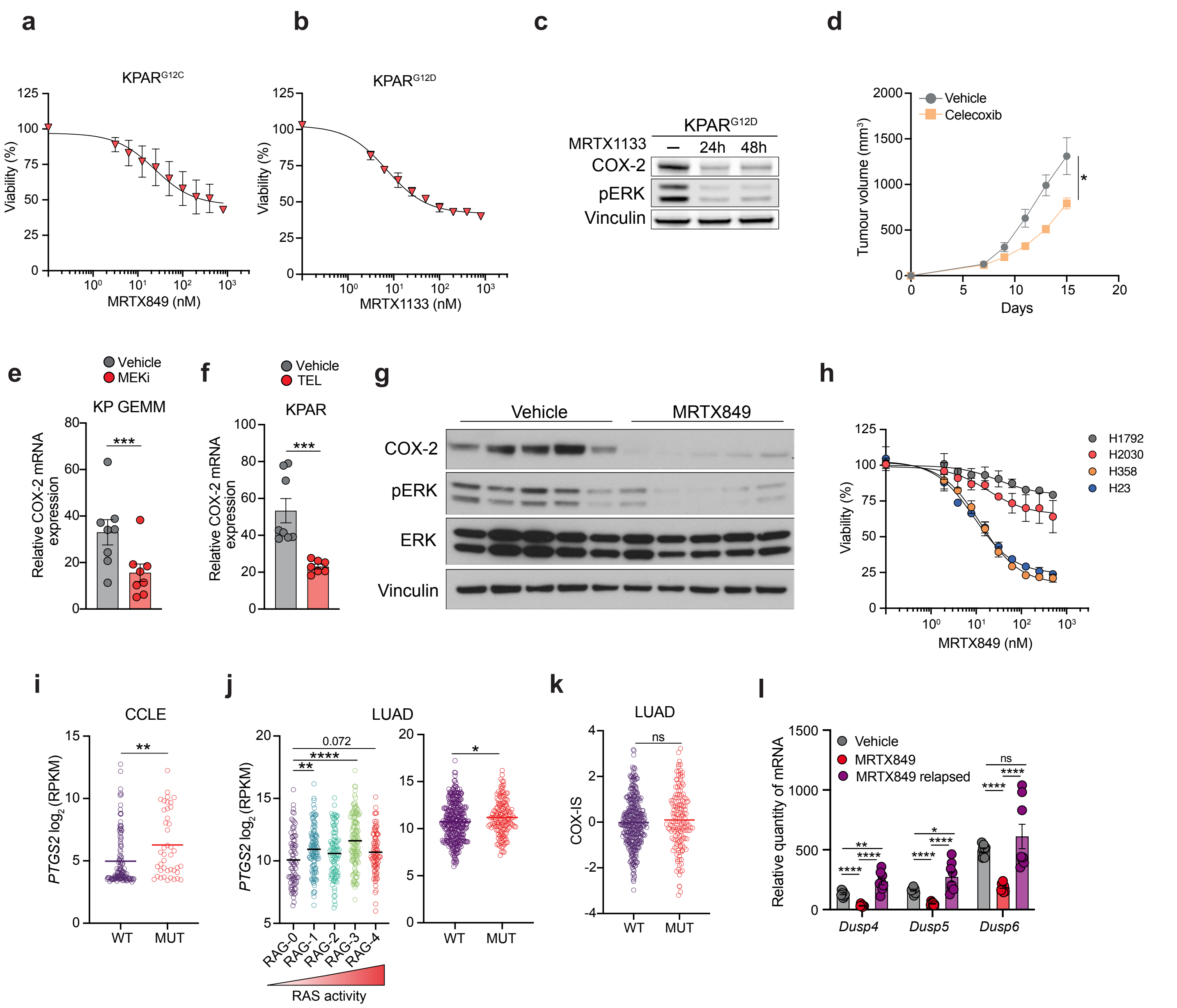

Supplementary Figure 9. Oncogenic KRAS drives tumour-intrinsic expression of COX-2 in LUAD
(A-B) Viability of KPAR^{G12C} cells treated with serial dilutions of MRTX849 (A) or KPAR^{G12D} cells treated with MRTX1133 (B) for 72h.
(C) Immunoblot for COX-2 in KPAR^{G12D} cells treated with 100nM MRTX1133 for 24h or 48h.
(D) Mean ± SEM 3LL ΔNRAS tumour volumes in mice treated with daily oral gavage of 30mg/kg celecoxib. Daily celecoxib treatment was initiated on day 7.
(E-F) COX-2 mRNA expression by qPCR in KP GEMM treated for 7d with 1.3mg/kg trametinib (MEKi) (B) or KPAR orthotopic tumours treated for 7d with 1.3mg/kg trametinib, 1.6mg/kg everolimus and 16.6mg/kg linsitinib (TEL) (C).
(G) Immunoblot for COX-2 in KPAR^{G12C} tumours treated for 7d with 50mg/kg MRTX849.
(H) Viability of human KRAS^{G12C} lung cancer cell lines treated with serial dilutions of MRTX849 for 72h.
(I) COX-2 expression in RAS-WT and RAS-mutant human lung cancer cell lines from the CCLE database.
(J) COX-2 mRNA expression in LUAD samples from TCGA stratified by RAS-activity (left) or RAS mutational status (right).
(K) COX-IS in RAS-WT and RAS-mutant LUAD samples from TCGA.
(L) mRNA expression of KRAS pathway targets in MRTX849 on-treatment and relapsed KPAR^{G12C} tumours.
For (D-F and I) data are mean ± SEM, n=7-9 per group. Samples were analysed using unpaired, two-tailed Student's t-test (E-F, I-J and K), one-way ANOVA, (J and L) FDR 0.05 or two-way ANOVA (D) ; ns, not significant, * P<0.05, ** P<0.01, *** P<0.001, **** P<0.0001.
